# Excess heat production of the pair annihilation of ionic vacancies in a copper redox reaction using a double bipolar MHD electrode

**DOI:** 10.1038/s41598-024-51834-w

**Published:** 2024-01-16

**Authors:** Makoto Miura, Atsushi Sugiyama, Yoshinobu Oshikiri, Ryoichi Morimoto, Iwao Mogi, Miki Miura, Yusuke Yamauchi, Ryoichi Aogaki

**Affiliations:** 1 Tohoku Polytechnic College, Kurihara, Miyagi 987-2223 Japan; 2 Yoshino Denka Kogyo, Inc., Yoshikawa, Saitama 342-0008 Japan; 3 Yamagata College of Industry and Technology, Matsuei, Yamagata, 990-2473 Japan; 4 Saitama Industrial Technology Center, Kawaguchi, Saitama 333-0844 Japan; 5 Institute for Materials Research, Tohoku University, Aoba-ku, Sendai, 980-8577 Japan; 6 Polytechnic Center Kimitsu, Kimitsu, Chiba 299-1142 Japan; 7 Australian Institute for Bioengineering and Nanotechnology (AIBN), The University of Queensland, Brisbane, QLD 4072 Australia; 8 Department of Materials Process Engineering, Graduate School of Engineering, Nagoya University, Nagoya, 464−8603 Japan; 9 Department of Chemical and Biomolecular Engineering, Yonsei University, 50 Yonsei-ro, Seodaemun-gu, Seoul, 03722 South Korea; 10 Polytechnic University, Sumida, Tokyo 130-0026 Japan

**Keywords:** Energy science and technology, Materials science, Nanoscience and technology, Physics

## Abstract

Through a copper double bipolar magnetohydrodynamic (MHD) electrode (MHDE) producing twice the amounts of ionic vacancies than a conventional single MHDE, the molar excess heat of the pair annihilation of ionic vacancies, 702 kJ mol^−1^ at 10 T on average was obtained in a copper redox reaction. It was about twice as large as that of a single MHDE, 387 kJ mol^−1^ at the same magnetic field. This result strongly suggests that a multi-channel bipolar MHDE will produce much greater excess heat. To conserve the linear momentum and electric charge during electron transfer in an electrode reaction, ionic vacancies are created, storing the solvation energy in the polarized core of the order of 0.1 nm, and the pair annihilation of the vacancies with opposite charges liberates the energy as excess heat. The promoted excess heat by the double bipolar MHDE with a diffuser at 10 T was 710 ± 144 kJ mol^−1^, whereas as mentioned above, 702 ± 426 kJ mol^−1^ was obtained by the same electrode without such a diffuser. From the theoretical excess heat of 1140 kJ mol^−1^, the collision efficiencies in pair annihilation were 0.623 ± 0.126 and 0.616 ± 0.374, respectively. From these results, the reproducibility of the thermal measurement was experimentally validated. At the same time, it was concluded that at magnetic fields beyond 10 T, the concentration of ionic vacancy and the collision efficiency take constant uppermost values.

## Introduction

Recently, in a copper redox reaction and a ferricyanide-ferrocyanide redox reaction under high magnetic fields, the excess heat as the reaction heat of the collision of ionic vacancies with opposite signs has been measured^1,2^. The former excess heat was 411 ± 280 kJ mol^−1^ in a 300 mol m^−3^ CuSO_4_ + 500 mol m^−3^ H_2_SO_4_ solution at 10 to 15 T, which is about 1.5 times larger than the molar combustion heat of hydrogen 286 kJ mol^−1^, whereas the latter was 22.4 ± 15.3 kJ mol^−1^ in a 300 mol m^−3^ equimolar K_3_[Fe(CN)_6_] + K_4_[Fe(CN)_6_] + 100 mol m^−3^ KCl solution at 10–15 T (See Table 1). Since the measured excess heat consists of the product of the collision efficiency by the solvation energy stored in a pair of ionic vacancies with opposite signs, such a large difference was attributed to the different collision efficiencies as well as different solvation energies. Namely, the former solvation energy is calculated to be 570 kJ mol^−1^, and the latter is 112 kJ mol^−1^. Therefore, the collision efficiencies result in 0.721 ± 0.491, and 0.200 ± 0.137.

**Table 1:** The molar excess heat ${\gamma }_{{\text{col}}}{Q}_{{\text{ann}}}$ obtained at 10–15 T in the previous papers^1,2^.

Electrode type	Copper redox reaction ${\gamma }_{{\text{col}}}{Q}_{{\text{ann}}}$ / kJ mol^−1^	Ferricyanide-Ferrocyanide redox reaction ${\gamma }_{{\text{col}}}{Q}_{{\text{ann}}}$ / kJ mol^−1^
Single MHDE	411 ± 280	22.4 ± 15.3

**Figure 1:**
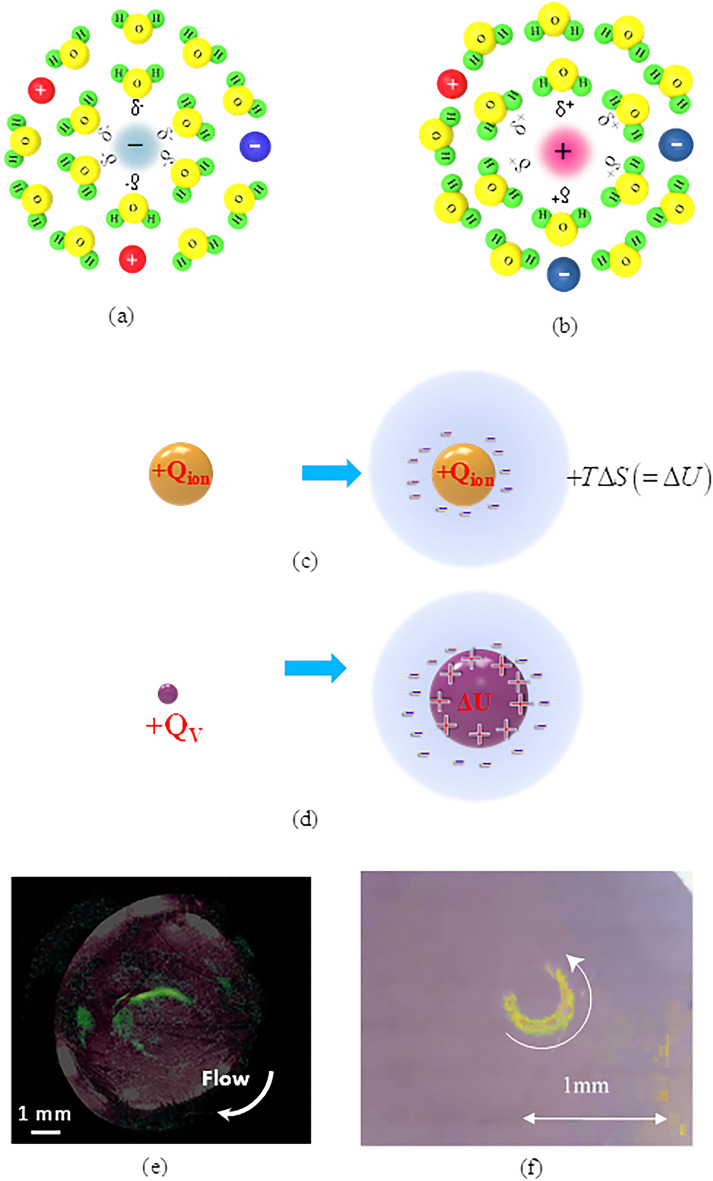
Solvation of embryo vacancy and formation of microbubble clusters. (**a**) Solvated negative vacancy. ${\delta }^{-}$: partial negative charge, ${\delta }^{+}$: partial positive charge, : anion,  cation, : hydrogen atom, : oxygen atom. (**b**) Solvated positive vacancy. (**c**) Solvation process of an isolated ion. ${+Q}_{ion}$: the positive charge of cation, $\Delta U$: the solvation energy, $T$: the absolute temperature, $\Delta S$: the produced entropy. (**d**) Solvation process of an embryo vacancy. ${+Q}_{V}$: the polarized positive charge of the positive embryo vacancy, $\Delta U$: the solvation energy. (**e**) Microbubble clusters observed in a copper cathodic deposition^7^. (**f**) Microbubble clusters observed in a copper anodic dissolution^8^.

In general, an ionic vacancy is created as a by-product of an electrode reaction. From the theoretical and experimental examinations, the following natures of ionic vacancies have been clarified; as shown in Fig. 1a,b, an ionic vacancy solvated in a solution is an electrically polarized free space of the order of 0.1 nm (vacancy core) surrounded by an ionic cloud with opposite charge, which is created initially in an electrode reaction as an embryo vacancy to keep the conservation of linear momentum and electric charge^3^. Negative and positive vacancies in Fig. 1a,b are created at the cathode and anode, respectively.

As shown in Fig. 1c, a charged particle such as an ion in a free space is energetically unstable among solvent molecules, so that it is immediately stabilized by solvation, releasing solvation energy as entropy. However, in the case of an embryo vacancy, as shown in Fig. 1d, using the solvation energy, the vacancy core is expanded to a size of the order of 0.1 nm instead of entropy production, so that the solvation energy is stored in the expanded core without any entropy production^4^. After attaining a natural lifetime of 1 s^5^, the vacancy is extinguished, following the same process in the reverse direction. An ionic vacancy hence acts as an iso-entropic particle without any interplay between solvent molecules. This suggests that an ionic vacancy moves like a gas molecule, and plays a role of an atomic-scale lubricant.

The existence of ionic vacancies in a solution has been experimentally ascertained by observing the clusters of microbubbles containing dissolved nitrogen gas in various electrode reactions under high magnetic fields^6–8^: In an electrode reaction under a vertical magnetic field, a tornado-like rotation called vertical MHD flow emerges over a disk electrode (vertical MHD electrode). Under the rotation, a radial flow toward the electrode center is secondarily induced, so that the electrode surface is covered with ionic vacancies, which form a vacancy layer. Due to the iso-entropic property of an ionic vacancy, the layer’s viscosity decreases to zero, so microscopic vortexes called micro MHD flows are induced. The upward flows of the vortexes swallow ionic vacancies, yielding nanobubbles after collisions of many vacancies at once^9,10^. As a nanobubble arises from the collisions of many vacancies^11^, it has many polarized electric charges on its surface, characterized by strong specific adsorption. The nanobubbles furthermore collide with each other in the rotation of the vertical MHD flow, finally forming microbubbles. In Fig. 1e,f, the observed microbubble clusters in copper cathodic deposition and copper anodic dissolution are represented^7,8^.

The chemical nature of the nanobubbles has been examined by the adsorption onto newly created copper nuclei, which, though no hydrogen evolution, resulted in obvious dendritic growth (Magneto-dendrite effect^12^). This implies that the nanobubbles specifically adsorb on a copper surface like hydrogen molecules from hydrogen ions^13,14^, and suppress three-dimensional (3D) copper nucleation. The most remarkable point of this effect is that the rising deposition current greatly shifts to the anodic side from the hydrogen evolution potential; so that in a high magnetic field, copper cathodic deposition progresses in the absence of hydrogen evolution even in a current much higher than the cathodic limiting-diffusion current.

As has been mentioned above, the collision between ionic vacancies with the same sign yields nanobubbles, whereas the collision between ionic vacancies with opposite signs brings a quite different result, i.e., the annihilation of the vacancies via charge neutralization of ionic clouds. At the same time, the solvation energies stored in their cores are liberated as excess heat. To measure the excess heat in electrochemical reactions, calorimetry experiments have already been performed in complex electrochemical cells such as lithium batteries^15,16^. In order to exactly measure the quantity of heat, it is indispensable to keep electrode systems isothermal. Although such a condition is often interfered with by the nonuniform temperature fields occurring in electrode systems, we can easily accomplish an isothermal state by the strong stirring effect using a solution flow induced by Lorentz force called MHD flow. Fortunately, in magnetoelectrochemistry, several useful tools have been already developed for reaction analyses in a magnetic field^17–24^. Fahidy commented on the MHD effect that an MHD flow decreases the thickness of a diffusion layer, enhancing mass transfer in an electrode reaction^21–23^. Olivier theoretically examined the MHD effect on microelectrodes^24–27^ and established electrochemical impedance spectroscopy in a magnetic field^26,28,29^. White investigated the MHD effect at ultra-micro-disk-electrodes in non-aqueous systems containing organic reactants^30–32^. Using the flow-visualization techniques in a magnetic field, Mutschke and co-workers examined electrodeposition in cuboid cells under magnetic fields accompanied by 3D convections affected by a gravitational field, which were compared with numerical simulations^33,34^. The contributions of the MHD effect to the phase compositions of composite metals have been investigated by many researchers (Oliver, Alemany, Daltin, Chopart, Hinds, Coey, Zabiński)^35–43^.

As a remarkable magnetoelectrochemical effect, we can cite the magneto-convection by the gradient field force under a heterogeneous magnetic field, which promotes the mass transfer process in an electrode reaction^44–46^. A superimposed Lorentz force donates more complicated effects to deposit patterns and compositions (Tschulik, Uhlemann, Mutshchke, Dunne, Coey)^47–51^.

Especially, an MHD flow under a parallel magnetic field is efficiently provided by an MHD electrode (MHDE)^52–54^. The velocity and concentration distributions of the electrode are reduced to simple equations of the velocity and limiting diffusion current, where agreement between theory and the experimental result is excellent^23^.

In the preceding papers^1,2^, using an MHDE called circulation-type (c-type) MHDE, we have succeeded in measuring the excess heat by the collision (i.e., pair annihilation) of positive and negative ionic vacancies created in a copper redox reaction and a ferricyanide-ferrocyanide redox reaction. The means to derive the excess heat from the experimental data were however different; the former was a curve-fitting method, where the theoretical equation of the temperature change measured with a current sweep was applied to experimental data for curve-fitting. The latter was called Joule’s heat capacity method, where a calculated heat capacity (Joule’s heat capacity) was plotted against a parameter (the square of the current divided by the temperature change). In the present paper, we represent that the data obtained by both methods for a copper redox reaction are in good agreement.

**Figure 2:**
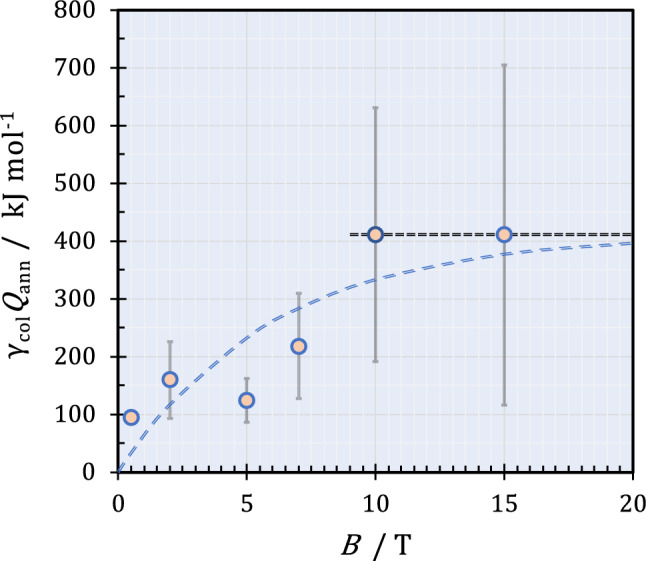
Plot of the measured excess heat ${\gamma }_{{\text{col}}}{Q}_{{\text{ann}}}$ versus magnetic flux density *B* by single c-type copper MHDEs ($m$ = 1) for a copper redox reaction in an acidic copper sulfate solution^1^. [CuSO_4_] = 300 mol m^−3^, [H_2_SO_4_] = 500 mol m^−3^.

Figure 2 exhibits the plot of the measured excess heat against magnetic flux density for a copper redox reaction in the preceding paper^1^. The measured excess heat increases with magnetic flux density, approaching a maximum constant value on average beyond 10 T. This means that the vacancy concentration also increases with the magnetic flux density, taking an uppermost value beyond 10 T. Therefore, to guarantee the accuracy and reproducibility of two different methods of excess heat at a constant collision efficiency, we should examine the response of the measured excess heat against the production quantity of ionic vacancies beyond 10 T.

In the present paper, first, we, therefore examine the mass balance of ionic vacancies in a c-type MHDE; and derive the concentration equation of ionic vacancy to obtain the largest constant value of the measured excess heat at a high magnetic field. Then, applying the curve-fitting and Joule’s heat-capacity methods to the same data of a copper redox reaction, we ensure the validity of both methods. Finally, to certify the reproducibility of Joule’s heat-capacity method, using a double bipolar MHDE, we measure the excess heat in a copper redox reaction; and compare it with the data obtained from a single MHDE. At the same time, to ascertain whether the mass balance of ionic vacancy attains a maximum state or not, we also attempt to measure the excess heat by the special double bipolar MHDE equipped with a diffuser extremely promoting the collision efficiency.

## Theory

**Figure 3:**
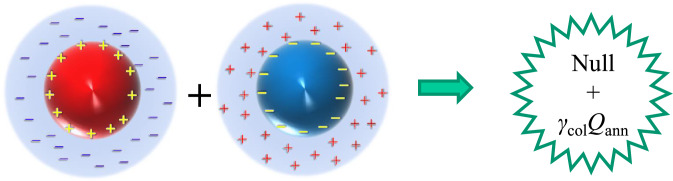
Collision of a pair of ionic vacancies with opposite signs. ${\gamma }_{{\text{col}}}{Q}_{{\text{ann}}}$, measured molar excess heat in case of a single MHDE ($m$ = 1).

As discussed above, the collisions of ionic vacancies with the same sign yield a nanobubble, whereas in the collision of ionic vacancies with opposite signs, as shown in Fig. 3, the stored solvated energy is released as excess heat. It has been successfully observed in a copper redox reaction^1^. Cathodic and anodic reactions of copper in a sulfuric acid solution involving ionic vacancies are expressed by

1 $${\text{Cu}}^{2 + } + 2{\text{e}}^{ - } \to {\text{Cu}} + {\text{V}}_{2 - } \;\;\;\left( {{\text{cathodic}}\;{\text{reaction}}} \right)$$

2 $${\text{Cu}} - 2{\text{e}}^{ - } \to {\text{Cu}}^{2 + } + {\text{V}}_{2 + } \;\;\;\left( {{\text{anodic}}\;{\text{reaction}}} \right)$$

where ${{\text{V}}}_{2-}$ and ${{\text{V}}}_{2+}$ are the ionic vacancies with two negative and positive unit charges, respectively, which are polarized from the conservation of electric charges in the electron transfer of the electrode reactions^3^, as will be shown in Eq. (3), they are neutralized in pair annihilation. In these reactions, two electrons transfer between the electrode and reactant, and the electric charges simultaneously move to/from the solution side, so that in case of the electron transfer from the electrode to the reactant (cathodic reaction), a vacancy with two polarized negative unit-charges ${{\text{V}}}_{2-}$ is created, while for the electron transfer from the reactant to the electrode, a vacancy with two polarized positive unit charges ${{\text{V}}}_{2+}$ emerges. When a pair of vacancies with opposite signs are annihilated by their collision, the stored solvation energy is released as follows.

3 $${{\text{V}}}_{2-}+{{\text{V}}}_{2+}\to {\text{Null}}+{\gamma }_{{\text{col}}}m{Q}_{{\text{ann}}}$$

where ${\gamma }_{{\text{col}}}m{Q}_{{\text{ann}}}$ is denoted as the measured molar excess heat (J mol^−1^), ${\gamma }_{{\text{col}}}$ is the collision efficiency, and $m$ is the number of the pair of cathode and anode of an MHDE. ${Q}_{{\text{ann}}}$ is the theoretical molar excess heat (solvation energy) (J mol^−1^). From Eq. (B11) in Supplement B, it is expressed by

4 $${Q}_{{\text{ann}}}=8\pi {N}_{{\text{A}}}\sigma {{R}_{{{\text{V}}}_{\alpha }}}^{2}$$

where ${N}_{{\text{A}}}$ is the Avogadro number, ${R}_{{{\text{V}}}_{\mathrm{\alpha }}}$ is the radius of the vacancy core (m) for $\alpha$ = 2 − or 2 + , $\sigma$ is the surface tension of water (7.2 × 10^–2^ J m^−2^) at 25 °C. In a 500 mol m^−3^ H_2_SO_4_ solution, as shown in Fig. C1, Supplement C, from the theoretical calculation based on the theory^4^, ${R}_{{{\text{V}}}_{\mathrm{\alpha }}}$ = 7.24 × 10^–10^ m (0.724 nm) is obtained. For a double MHDE ($m$ = 2) in the present case, by Eq. (3), the theoretical molar excess heat $m{Q}_{{\text{ann}}}$ in a 500 mol m^−3^ H_2_SO_4_ solution becomes 1140 kJ mol^−1^, which is twice as much as that of a single MHDE ($m$ = 1) 570 kJ mol^−1^ (See Fig. C1).

**Figure 4:**
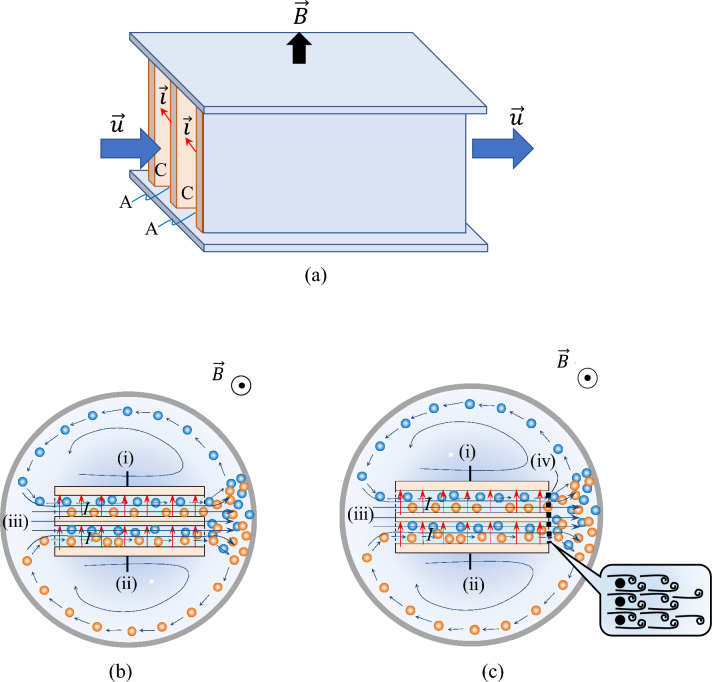
Double c-type bipolar MHDE. (**a**) A double bipolar MHDE. $\overrightarrow{B}$: magnetic flux density, $\overrightarrow{u}$: velocity, $\overrightarrow{i}$: current density, C: cathode, A: anode. (**b**) A bird’s-eye view of a double c-type bipolar MHDE. (**c**) A bird’s-eye view of a double c-type bipolar MHDE with a diffuser. (i): cathode, (ii): anode, (iii): MHD flow, (iv): diffuser (plastic net). : negative vacancy, : positive vacancy, : cross section of the wire of net, curled lines in the magnified view in Fig. 4c: Kármán vortexes.

In the preceding papers^1,2^, as mentioned above, for ionic vacancies with opposite signs to collide, a single c-type MHDE was used, which was composed of a channel with two open ends, where on the inner walls, a copper cathode and a copper anode of the same size were embedded facing each other. Ionic vacancies with opposite signs created on the cathode and anode are quickly conveyed by the MHD flow induced by Lorentz force and mixed to collide in front of the inner wall of the electrolysis cell. Due to the narrow vessel, the remainder of the vacancies escaping from the collisions can circulate for the next collisions. The excess heat generated by the collisions is measured by a thermal sensor attached to the inner wall of the vessel. Figure 4a exhibits a double c-type bipolar MHDE, where another copper plate is settled as a bipolar electrode in between the original anode and cathode. When an electrolytic current flows, as shown in Fig. 4b, both surfaces facing the cathode and anode act as an anode and a cathode, respectively. As a result, the electrode area becomes twice as large as that of a single MHDE, so that the produced amount of ionic vacancy becomes also twice, i.e., in Eq. (3), $m$ = 2 is introduced. Therefore, if the measured excess heat were twice, the reproducibility of this measurement would be ascertained. In Fig. 4c, a double bipolar MHDE equipped with a diffuser is represented. The diffuser is made of a plastic net with a 2.5 mm mesh weaved by 0.3 mm diameter wires, set in front of the outlet of the electrode channel. When an MHD flow slips through the mesh, it is violently stirred by Kármán vortexes, promoting collision efficiency. If the measured reaction heat were of the same level as that of an MHDE without such a diffuser, we could conclude that the collisions of ionic vacancies attain the uppermost level.

As shown in Eq. (A7), Supplement A, in steady state, the average vacancy concentration ${\overline{C}}_{{{\text{V}}}_{\mathrm{\alpha }}}$ in the reaction field settled in front of the inner wall of the electrolysis cell is determined by the average solution velocity $\overline{u }$ and the concentration difference of vacancy $\Delta {C}_{{{\text{V}}}_{\mathrm{\alpha }}}$ arising from vacancy extinction during the transfer in the reaction field.

A7 $${\overline{C}}_{{{\text{V}}}_{\mathrm{\alpha }}}=\frac{{\tau }_{{\text{eff}}}}{L}\overline{u} \Delta {C }_{{{\text{V}}}_{\mathrm{\alpha }}}$$

where $L$ is denoted as the *x* length of the reaction field; and ${\tau }_{{\text{eff}}}$ is the effective extinction period of vacancy defined by

A6 $$\frac{1}{{\tau }_{{\text{eff}}}}\equiv \frac{1}{{\tau }_{{\text{col}}}}+\frac{1}{{\tau }_{{\text{nano}}}}+\frac{1}{{\tau }_{{\text{life}}}}$$

where ${\tau }_{{\text{col}}}$ is denoted as the collision period of the ionic vacancy, ${\tau }_{{\text{nano}}}$ is the period of the multiple collision of ionic vacancies to a nanobubble, and ${\tau }_{{\text{life}}}$ is the natural lifetime of an ionic vacancy. ${\tau }_{{\text{eff}}}$ is therefore controlled by the smallest period, i.e.,${\tau }_{{\text{col}}}.$

In the same electrode system using the same current sweep rate, the parameters ${\tau }_{{\text{eff}}}$ and $L$ are regarded constant, and the average velocity $\overline{u}$ induced by Lorentz force increases with the applied magnetic field in the same current range. The more the average velocity $\overline{u}$ is enhanced, the shorter the staying period of ionic vacancies in the reaction field becomes, so that the chance of extinction by the collisions as well as the resultant concentration difference decreases. Namely, as the magnetic field increases, balancing with the increasing velocity $\overline{u}$, the concentration difference of vacancy $\Delta {C}_{{{\text{V}}}_{\mathrm{\alpha }}}$ decreases. Equation (A7) would therefore lead to a constant vacancy concentration ${\overline{C}}_{{{\text{V}}}_{\mathrm{\alpha }}}.$ In a sufficiently high magnetic field of more than 10 T, as has been shown in Fig. 2, the collision process of ionic vacancies is promoted to the maximum level, so that the vacancy concentration would also take the highest constant value. Such expectation will be verified by the uppermost promotion of the collision provided by an MHDE with a diffuser shown in Fig. 4c.

The measurement of the temperature difference is carried out by current sweeping. In the preceding paper on a copper redox reaction^1^, the excess heat was obtained by fitting a theoretical equation to experimental results. On the other hand, in the following paper on a ferricyanide-ferrocyanide redox reaction^2^, as a more precise method, Joule’s heat capacity method was adopted. In the present paper, applying both methods to the same experimental result obtained from an ordinary single MHDE, we examine the agreement of the values measured by both methods. The procedures for measuring the excess heat are simply elucidated as follows.

1) The curve-fitting method.

The cell voltage of the single MHDE $\Delta V$ (V) is measured with the increasing electrolytic current $I$ (A) with time $t$ (s), i.e.,

5 $$I=at$$

where $a$ is the sweep rate (A s^−1^). In the initial stage, the cell voltage $\Delta {V}_{1}$ (V) rapidly changes with the current $I$. However, beyond a critical current ${I}_{{\text{C}}}$ (A) for measurement, the cell voltage $\Delta {V}_{2}$ (V) is effectively expressed by the reaction overpotential $\Delta {V}_{{\text{react}}}$ and the ohmic drop ${R}_{{\text{sol}}}I$, i.e.,

6 $$\Delta {V}_{2}=\Delta {V}_{{\text{react}}}+{R}_{{\text{sol}}}I \quad \mathrm{for }\quad I\ge {I}_{{\text{C}}}$$

where ${R}_{{\text{sol}}}$ is the solution resistance $\left(\Omega \right)$.

Then, according to the preceding paper^1^, after compensating for the escaping heat from the electrolysis cell, the resulting compensated temperature difference $\Delta {T}^{*} ({\text{K}})$ between the solution and the magnet bore is expressed by the following 3rd-order equation of the current $I$.

7 $$\Delta {T}^{*}={a}_{0}+{a}_{2}{I}^{2}+{a}_{3}{I}^{3} \quad \mathrm{for } \quad I\ge {I}_{{\text{C}}}$$

where the coefficients ${a}_{3}, {a}_{2}$ and ${a}_{0}$ are expressed as follows.

8 $${a}_{3}\equiv \frac{{R}_{{\text{sol}}}}{3a{C}_{{\text{sys}}}}$$

9 $${a}_{2}\equiv \frac{1}{2a{C}_{{\text{sys}}}}\left(\Delta {V}_{{\text{react}}}+\frac{-T{\Delta }_{{\text{R}}}{S}_{{\text{R}}}+{\gamma }_{{\text{col}}}m{Q}_{{\text{ann}}}}{nF}\right)$$

10 $${a}_{0}\equiv \frac{1}{a{C}_{{\text{sys}}}}\left({\int }_{0}^{{I}_{{\text{C}}}}\Delta {V}_{1}I{\text{d}}I-\frac{\Delta {V}_{{\text{react}}}}{2}{I}_{{\text{C}}}^{2}+\frac{{R}_{{\text{sol}}}}{3}{I}_{{\text{C}}}^{3}\right)+\Delta {T}_{0}^{*}$$

where ${C}_{{\text{sys}}}$ is the calorimeter constant (J K^−1^), $\Delta {T}_{0}^{*}$ is the initial value of $\Delta {T}^{*}$ (K), $n$ is the positive charge number transferring in the cell reaction, and $F$ is the Faraday constant (96,500 C mol^−1^). $T$ is the absolute temperature of the system (K), ${\Delta }_{{\text{R}}}{S}_{{\text{R}}}$ is the molar entropy change in the cell reaction (J K^−1^ mol^−1^).

After analyzing the linear regression by Eq. (7) for the second range of the current larger than the critical current for measurement, i.e., $I$ ≥ ${I}_{{\text{C}}}$, the calorimeter constant ${C}_{{\text{sys}}}$ is first determined by the coefficient in Eq. (8).

11 $${C}_{{\text{sys}}}=\frac{{R}_{{\text{sol}}}}{3a{a}_{3}}$$

Then, the measured molar excess heat ${\gamma }_{{\text{col}}}m{Q}_{{\text{ann}}}$ is obtained from the coefficient ${a}_{2}$ in Eq. (9).

12 $${\gamma }_{{\text{col}}}m{Q}_{{\text{ann}}}=nF\left(2a{a}_{2}{C}_{{\text{sys}}}-\Delta {V}_{{\text{react}}}\right)+T{\Delta }_{{\text{R}}}{S}_{{\text{R}}}$$

For a redox cell reaction such as the present copper reaction, due to no chemical production, $T{\Delta }_{{\text{R}}}{S}_{{\text{R}}}$ = 0 is assumed.

2) The Joule’s heat capacity method.

The excess heat is produced by the collisions of ionic vacancies creating with the current sweep in Eq. (5). In the low current range, due to weak Lorentz force, the collision of vacancy hardly occurs, whereas in the high current range, Joule’s heat prevails over the excess heat. This means that except for the middle current range, we cannot measure the excess heat correctly^2^.

We first draw the locus of Joule’s heat capacity ${R}_{{\text{J}}}\left(I\right)$ defined by

13 $${R}_{{\text{J}}}\left(I\right)\equiv {Q}_{{\text{Joule}}}/\Delta {T}^{*}$$

against the parameter* x* expressed by

14 $$x\equiv {I}^{2}/\Delta {T}^{*}$$

where $\Delta {T}^{*}$ is the compensated temperature difference (K), and ${Q}_{{\text{Joule}}}$ is the Joule’s heat (J mol^−1^) calculated by the following equation.

15 $${Q}_{{\text{Joule}}}=\frac{1}{a}{\int }_{0}^{I}\Delta VI{\text{d}}I$$

where $\Delta V$ is the cell voltage (V) including $\Delta {V}_{1}$ and $\Delta {V}_{2}$ in the curve fitting method. Then, the following equation is applied to the linear portion of the locus with a negative slope $-{b}_{{\text{mid}}}$ (J A^−2^) emerging in the middle current range.

16a $${R}_{{\text{J}}}\left(I\right)={C}_{{\text{sys}}}-{b}_{{\text{mid}}}x$$

where ${C}_{{\text{sys}}}$ is the calorimeter constant in the middle current range (J K^−1^), and the coefficient ${b}_{{\text{mid}}}$ is defined by

16b $${b}_{{\text{mid}}}\equiv \frac{{\gamma }_{{\text{col}}}m{Q}_{{\text{ann}}}}{2nFa}$$

From the linear regression, the coefficient ${b}_{{\text{mid}}}$ is determined, so that the excess heat is calculated by the following equation.

17 $${{\gamma }_{{\text{col}}}m{Q}_{{\text{ann}}}=2nFab}_{{\text{mid}}}$$

In a ferricyanide-ferrocyanide redox reaction, as the current further increases, the excess heat is overwhelmed by Joule’s heat. As a result, in the high current range, the Joule’s heat capacity is kept constant, drawing a level line in the locus.

18 $${R}_{{\text{J}}}\left(I\right)={C}_{{\text{sys}}}^{*}$$

where ${C}_{{\text{sys}}}^{*}$ is the calorimeter constant (J K^−1^) in the high current range. In the high current range, due to violent stirring of the MHD flow, molecular mixing is completed, so that by measuring it under the various amounts of water, the certification of this method was successfully carried out^2^.

## Discussion

**Figure 5:**
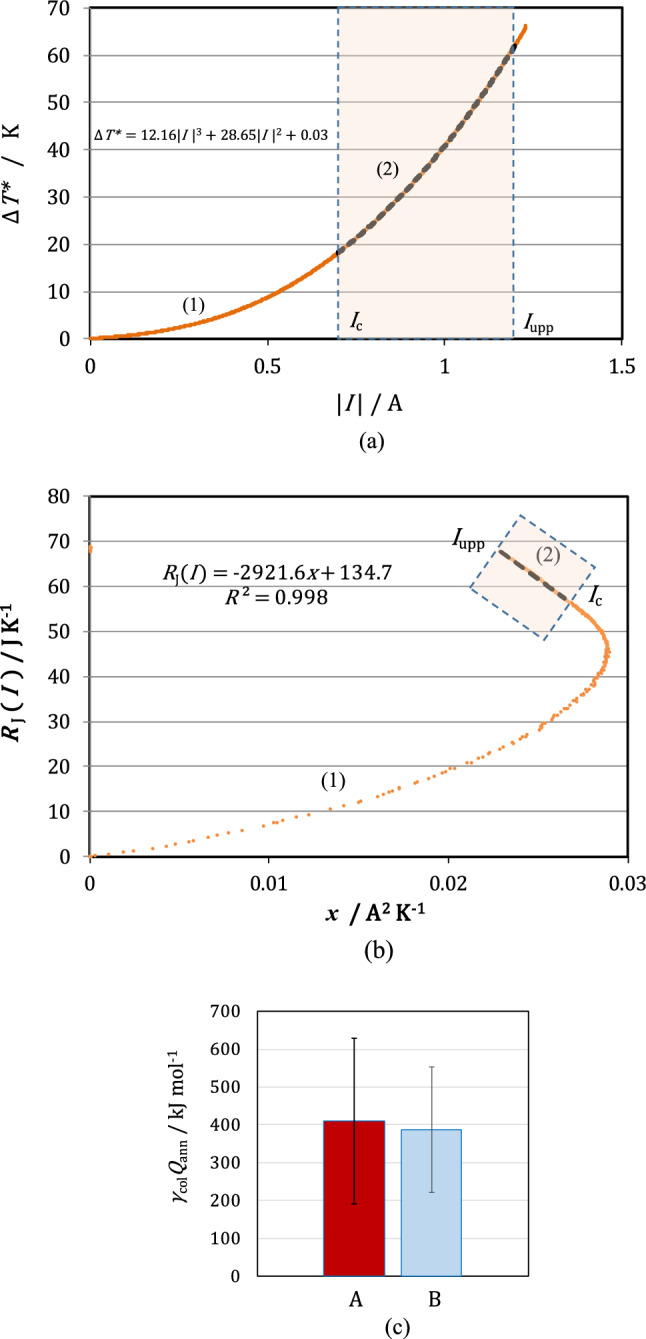
Comparison of the plots between the curve-fitting method and the Joule’s heat capacity method by a single c-type MHDE ($m$ = 1) at 10 T in a copper redox reaction. (**a**) The plot of the curve-fitting method. Orange solid line: the plot of the experimental data, black break line: the plot of the curve fitting, $\Delta {T}^{*}$: the compensated temperature difference between the solution and the magnet bore, $I$: the sweeping current. ${I}_{{\text{c}}}$: the critical current, ${I}_{{\text{upp}}}$: the upper limit of the current. The numbers (1) and (2) in Fig. 5a indicate low and medium current ranges. The excess heat obtained from the range (2) is 190 kJ mol^−1^. (**b**) The locus of the Joule’s heat capacity method. ${R}_{J}\left(I\right)$: the Joule’s heat capacity defined by Eq. (13), $x$: the parameter defined by Eq. (14). The numbers (1) and (2) correspond to the same low and middle current ranges as the ranges (1) and (2) in Fig. 5a, respectively. The value determined by the range (2) is 226 kJ mol^−1^. (**c**) Comparison of the measured excess heat ${\gamma }_{{\text{col}}}{Q}_{{\text{ann}}}$ between the curve-fitting method and the Joule’s heat capacity method. A: The molar excess heat determined by the curve-fitting method (411 ± 219 kJ mol^−1^). B: The molar excess heat by the Joule’s heat capacity method (387 ± 165 kJ mol^−1^). The solution is composed of copper sulfate and sulfuric acid. [CuSO_4_] = 500 mol m^−3^, [H_2_SO_4_] = 300 mol m^−3^.

Figure 5a shows an example of the application of the curve-fitting method to the plot of the temperature difference $\Delta {T}^{*}$ against the current $I$ obtained by a single c-type MHDE at 10 T, where the temperature difference attains an upper limit of 60 K, and the theoretical curve fits well with the experimental data. To guarantee the accuracy of the coefficients ${a}_{3}$ and ${a}_{2}$ of the 3rd and 2nd powers of the current $I$ in Eq. (7), it is important to take a sufficient current range for curve-fitting as wide as possible. As a result, though much smaller than the average value shown in Fig. 5c, the excess heat in this case was determined 190 kJ mol^−1^.

In Fig. 5b, by the procedure of the Joule’s heat capacity method mentioned above, using the same data, we drew the locus of the Joule’s heat capacity ${R}_{{\text{J}}}\left(I\right)$ defined by Eq. (13) against the parameter *x* in Eq. (14). Then, Eq. (16a) was applied to the linear portion of the locus with a negative slope in the middle current range. In the present case of copper redox reaction, due to a large amount of excess heat production, because Joule’s heat could not surpass the excess heat even in the high current range, instead of the locus of a level line, only a wide middle current range appeared. From the experimental result, the average excess heat measured by the Joule’s heat capacity method was determined to be 226 kJ mol^−1^, which, in view of the experimental errors of the order of 100 kJ mol^−1^ shown in Fig. 5c, agreed well with 190 kJ mol^−1^ by the curve-fitting method.

In a ferricyanide-ferrocyanide redox reaction, since the excess heat produced in a single MHDE is not so large that the temperature difference $\Delta {T}^{*}$ only attains an upper limit of several degrees. Such small excess heat is easily surpassed by Joule’s heat, so that in the high current range, the locus of a level line of ${C}_{{\text{sys}}}^{*}$ emerges from the dominant Joule’s heat. Due to the presence of the level line, the wide current range to warrant the sufficient accuracy for the curve-fitting method is hardly provided. This is the reason why Joule’s heat capacity method was adopted in the preceding paper on a ferricyanide-ferrocyanide redox reaction^2^.

Figure 5c exhibits the comparison of the excess heat many times measured by both methods, where a lot of copper single MHDEs were used at a magnetic flux density of 10 T. As shown in Fig. 5c, though scattering, both methods lead to almost the same average values, i.e., for the curve-fitting method, ${\gamma }_{{\text{col}}}{Q}_{{\text{ann}}}$ = 411 ± 219 kJ mol^−1^ and for the Joule’s heat capacity method, ${\gamma }_{{\text{col}}}{Q}_{{\text{ann}}}$ = 387 ± 165 kJ mol^−1^. Though almost the same results are obtained, Joule’s heat capacity method is superior to the curve-fitting method, because it is applicable to all the cases.

**Figure 6:**
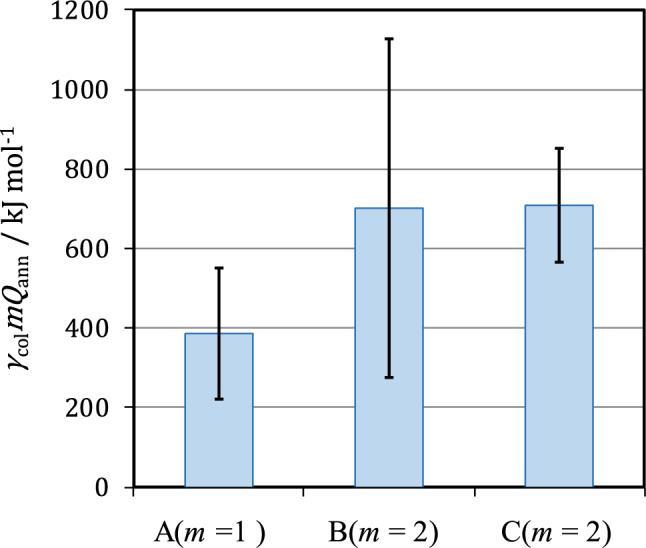
Comparison between the excess heats measured by the single and double MHDEs at 10 T. A: Measured excess heat by the single c-type MHDE (387 ± 165 kJ mol^−1^), B: Measured excess heat by the double c-type bipolar MHDE (702 ± 426 kJ mol^−1^), C: Measured excess heat by the double c-type bipolar MHDE with a diffuser (710 ± 144 kJ mol^−1^). ${\gamma }_{{\text{col}}}{mQ}_{{\text{ann}}}$, the measured excess heat, where *m* = 1 is applied to the single c-type MHDE, and* m* = 2 is to the double c-type bipolar MHDEs. The calculation was performed by the Joule’s heat capacity method. The solution is composed of copper sulfate and sulfuric acid. [CuSO_4_] = 300 mol m^−3^, [H_2_SO_4_] = 500 mol m^−3^.

Furthermore, to certify the reproducibility of Joule’s heat capacity method by controlling the production amount of ionic vacancy, the excess heat at 10 T was measured by the double bipolar MHDE shown in Fig. 4a. The actual calculation procedure of the obtained data is exhibited in Supplement D. As shown in Fig. 6, the measured excess heat by the single MHDE was ${\gamma }_{{\text{col}}}m{Q}_{{\text{ann}}}$ = 387 ± 165 kJ mol^−1^ ($m$ = 1) whereas for the double bipolar MHDE, we obtained ${\gamma }_{{\text{col}}}m{Q}_{{\text{ann}}}$ = 702 ± 426 kJ mol^−1^ ($m$ = 2), which was about twice as much as that of the single one. This result validates the sufficient reproducibility of Joule’s heat capacity method. At the same time, this result indicates that the collision efficiencies in both cases agree well with each other, having a constant value of around 0.6. Figure 6 represents that the measured excess heat increases with $m,$ i.e., the number of the pair of cathode and anode of an MHDE. Using a multi-channel bipolar MHDE system, we could make a new device to produce greater excess heat. If it is so, the temperature of the solution will highly increase up to a boiling point. In such a case, whether an ionic vacancy is destroyed or kept stable? The answer is that since the microscopic structure of an ionic vacancy is, as shown in Supplement C, strongly constructed by the Coulomb force of an ionic cloud formed by solvation, it remains stable even at a boiling point with other solvated ions with the same structures of ionic clouds.

To ascertain that the measured excess heat corresponds to the maximum consumption of the vacancies by the collisions, a double bipolar MHDE equipped with a diffuser shown in Fig. 4c was used in the same experiment at 10 T. As shown in Fig. 6, using Joule’s heat capacity method, the measured excess heat was determined ${\gamma }_{{\text{col}}}m{Q}_{{\text{ann}}}$ = 710 ± 144 kJ mol^−1^. As has been discussed in Eq. (4), in the present case, the theoretical excess heat $m{Q}_{{\text{ann}}}$ = 1140 kJ mol^−1^ was derived, so the collision efficiency in this case was calculated by ${\gamma }_{{\text{col}}}$ = 0.623 ± 0.126, whereas ${\gamma }_{{\text{col}}}$ = 0.616 ± 0.374 was obtained for the measured excess heat 702 ± 426 kJ mol^−1^ of the double bipolar MHDE without such a diffuser, which are summarized in Table 2. Namely, whether the diffuser was present or not, almost the same excess heat was obtained. This implies that in the copper redox reaction, the collision efficiency ${\gamma }_{{\text{col}}}$ takes a constant value around 0.6 in the measurements of single and double MHDEs even beyond 10 T. Namely, the mass balance of ionic vacancy attains an uppermost state, providing a constant vacancy concentration as well as a constant collision efficiency. The reason why the collision efficiency ${\gamma }_{{\text{col}}}$ = 1.0 cannot be attained even in the highest state may be attributed to the losses by the natural extinction of ionic vacancies with a lifetime of 1 s and the conversion to nanobubbles.

**Table 2:** The molar excess heat ${\gamma }_{{\text{col}}}{mQ}_{{\text{ann}}}$ and collision efficiency ${\gamma }_{{\text{col}}}$ newly observed.

Electrode type	Copper redox reaction without a diffuser ${\gamma }_{{\text{col}}}{mQ}_{{\text{ann}}}$ / kJ mol^−1^	Copper redox reaction with a diffuser ${\gamma }_{{\text{col}}}{mQ}_{{\text{ann}}}$ / kJ mol^−1^
Single MHDE (*m* = 1)	387 ± 165	–
	(${\gamma }_{{\text{col}}}$ = 0.679 ± 0.289)	–
Double bipolar MHDE (*m* = 2)	702 ± 426	710 ± 144
	(${\gamma }_{col}$ = 0.616 ± 0.374)	(${\gamma }_{{\text{col}}}$ = 0.623 ± 0.126)

In conclusion, the average excess heat in a copper redox reaction measured by the bipolar double MHDE attained up to 702 kJ mol^−1^ on average at 10 T, which was about twice as much as that of the single MHDE 387 kJ mol^−1^. This validates the reproducibility of the measurement of the excess heat production in the copper redox reaction. Since in the experiment where the collision efficiency of ionic vacancy was ultimately enhanced by a diffuser, we obtained almost the same excess heat on average (710 kJ mol^−1^), it was ascertained that in this system, the collision efficiency attains the upper limit of around 0.6 beyond 10 T, and the vacancy concentration is balanced by creation and extinction, reaching a constant value beyond 10 T. However, in the present paper, the highest values of the vacancy concentration and collision efficiency have not yet been obtained, so in the following paper, we will present the experimental results of the excess heat measurement of a ferricyanide and ferrocyanide redox reaction by double and triple bipolar-MHDEs, clarifying the universality of the highest vacancy concentration as well as the highest collision efficiency. Anyway, through multi-channel bipolar MHDEs, we could utilize much greater excess heat in electrochemical reactions. This is unquestionably quite important and useful for recovering the abandoned heat in electrochemical industries.

## Method

The copper deposition was carried out in a 300 mol m^−3^ CuSO_4_ + 500 mol m^−3^ H_2_SO_4_ solution. Water was prepared by a pure water production system (@Milli-Q, Merck Millipore). CuSO_4_ and H_2_SO_4_ were in analytical grade (Fujifilm Wako Pure Chemical Co.). A single MHDE and a double bipolar MHDE were composed of channels of acrylic acid resin with two open ends, respectively; the channels were 10 mm high, 5 mm wide, and 22 mm long. For the single MHDE, a pair of rectangular Cu electrodes (10 × 20 × 1 mm, Iwasaki Co., oxygen-free copper, 99.96% purity) working as a cathode and an anode were embedded on the inner side walls. For the double bipolar MHDE, another copper plate of the same size was inserted as a bipolar electrode between the outer electrodes. After confirming that the influence of a magnetic field up to 15 T was below the environmental thermal disturbance, two thermal sensors (T-type thermocouple) were attached to the electrodes from the outside of the channel, whose leads were connected to a measuring instrument (KEYENCE Co., NR-600 with NR-TH08 unit). Then, the single or double MHDE was set in a vessel containing an electrolyte solution of 20.0 cm^3^, and the whole electrode system was settled in the bore space of the 15 T-cryocooled superconducting magnet at the High Field Laboratory for Superconducting Materials, Institute for Materials Research, Tohoku University. The solution flows in the single and double MHDEs were optically observed by a microscope (AnMo Electronics Co., Dino-Lite Premier2 S-DINOAD7013MT) from the bottom of the bore. Finally, for each MHDE, other two sensors were inserted to monitor the temperatures of the solution and the bore space. After ascertaining that the sensors attached to the electrodes indicated the same temperature as that of the solution, for simplicity, they were removed from the electrodes. The two sensors in the solution and the bore space were used for measurement. For each case, sweeping the electrolysis current *I* in a rate of 0.2 mA s^−1^ from 0 to 1.2 A with a potentiostat (Toho Technical Research Co., Ltd., PS-2000) in galvanostatic mode, we measured the potential response $\Delta V$ between the outer cathode and anode of each MHDE. The electrode potentials of the cathode and anode were measured by the tentative reference electrode of a copper rod of 0.3 mm diameter. During the experiment, the temperatures of the electrode, the solution, and the bore space were measured. After attaining an upper limit of 1.2 A, to measure the heat escaping from the electrode system, the current was switched off, and the decreasing temperature of the solution was recorded by a personal computer.

Finally, to ascertain whether the collision efficiency of ionic vacancy attains an upper limit at 10 T, we measured the excess heat production by using a double bipolar MHDE equipped with a plastic net with 2.5 mm mesh weaved by 0.3 mm wires at the outlet of the channel as shown in Fig. 4c.

### Supplementary Information

Supplementary Information.

## Data Availability

Supplement D is a file explaining the handling of raw data. The datasets used and/or analysed during the current study available from the corresponding author on reasonable request.
