# Supplementary material for: Excess heat production of the pair annihilation of ionic vacancies in a copper redox reaction using a double bipolar MHD electrode
Source: Sci Rep. 2024 Jan 16;14:1424. doi: 10.1038/s41598-024-51834-w (PMC10792075; doi:10.1038/s41598-024-51834-w)
Supplement: Supplementary file 1 — Supplementary Information. [file 41598_2024_51834_MOESM1_ESM.pdf]

Supplementary information (A, B, C, D)

Excess Heat Production of the Pair Annihilation of Ionic Vacancies in a Copper Redox Reaction using a Double Bipolar MHD Electrode

Makoto Miura [1*], Atsushi Sugiyama [2], Yoshinobu Oshikiri [3], Ryoichi Morimoto [4], Iwao Mogi [5], Miki Miura [6], Yusuke Yamauchi [7, 8, 9*], Ryoichi Aogaki [10*]

1 Tohoku Polytechnic College, Kurihara, Miyagi 987-2223, Japan
2 Yoshino Denka Kogyo, Inc., Yoshikawa, Saitama 342-0008, Japan
3 Yamagata College of Industry and Technology, Matsuei, Yamagata 990-2473, Japan
4 Saitama Industrial Technology Center, Kawaguchi, Saitama 333-0844, Japan
5 Institute for Materials Research, Tohoku University, Aoba-ku, Sendai 980-8577, Japan
6 Polytechnic Center Kimitsu, Kimitsu, Chiba 299-1142, Japan
7 Australian Institute for Bioengineering and Nanotechnology (AIBN), The University of Queensland, Brisbane, QLD 4072, Australia
8 Department of Materials Process Engineering, Graduate School of Engineering, Nagoya University, Nagoya 464−8603, Japan.
9 Department of Chemical and Biomolecular Engineering, Yonsei University, 50 Yonsei-ro, Seodaemun-gu, Seoul 03722, South Korea
10 Polytechnic University, Sumida, Tokyo 130-0026, Japan

Corresponding authors
Correspondence to Makoto Miura (email: miura.makoto@tohoku-pc.ac.jp) or Ryoichi Aogaki (email: ryoaochan@aol.com) or Yusuke Yamauchi (email: y.yamauchi@uq.edu.au)

Supplement A. Derivation of a mass balance equation of ionic vacancy in steady state

In front of the inner wall of the electrolysis cell, as shown in Fig. A1, let us first assume a polygon as a reaction field of ionic vacancy connecting the electrode area and the front area of the inner wall of the vessel, so the back edges of the electrodes are located at $x = 0$, and the

portion in front of the inner wall is defined at $x = L$. The vacancies with opposite signs producing at the electrode area are conveyed by the MHD flow induced by Lorentz force to collide with each other on the way to the front of the inner wall. The vacancies escaping the collisions circulate the vessel, returning to the electrode area. The decreased concentration of the returning vacancies recovers its initial value $C_{\mathrm{V}_\alpha,\mathrm{elec}}$ at $x = 0$. Here, for simplicity, the concentration is assumed constant in the z-direction. Then, the mass balance of ionic vacancy in the *x*-direction within a parallelepiped with a unit length in the *y*-direction (backward of this paper) is considered; planes 12 and 43 are separately placed each other with a distance d*x*, whereas plane 23 is located ahead from the plane 14 at a distance of h in the *z*-direction.

The quantity of ionic vacancies conveyed by the fluid flow through the area $1 \times h$ of the plane 12 per unit time is expressed by $\int_0^h C_{\mathrm{V}_\alpha} \bar{u} \mathrm{d}z$, where C_{V_α} is the concentration of ionic vacancy with α-unit-charges including sign, and $\bar{u}$ is the average velocity of the solution, respectively. The difference in the amount of vacancies coming through plane 12 and leaving from plane 43 is given by

$$-\mathrm{d}x \frac{\partial}{\partial x} \int_0^h C_{\mathrm{V}_\alpha} \bar{u} \mathrm{d}z \ \ (< 0) \qquad \text{(A1)}$$

In this region, the ionic vacancies are consumed by collisions, so that the mass balance becomes negative.

Inside the polygon, the ionic vacancies with opposite signs collide with each other to neutralize their charges, releasing their stored solvation energies. The losing amount in the parallelepiped per unit time is represented by

$$-\mathrm{d}x \int_0^h \frac{C_{\mathrm{V}_\alpha}}{\tau_{\mathrm{col}}} \mathrm{d}z \ \ (< 0) \qquad \text{(A2)}$$

where τ_{col} is the collision period of the vacancy.

The vacancies are also consumed by the collisions between the vacancies with the same sign. Due to electrostatic repulsion, their 1-1 collision does not occur, but occurs in an upward micro MHD flow with other several vacancies, yielding a nanobubble [9, 10]. The losing quantity of vacancies per unit time in this process is therefore expressed by

$$-\mathrm{d}x \int_0^h \frac{C_{\mathrm{V}_\alpha}}{\tau_{\mathrm{nano}}} \mathrm{d}z \ \ (< 0) \qquad \text{(A3)}$$

where τ_{nano} is denoted as the period of the multiple collision of ionic vacancies to a nanobubble.

An ionic vacancy has a natural lifetime of about 1 s [5], so the amount of the extinguishing vacancies is also expressed by

$$-\mathrm{d}x \int_0^h \frac{C_{\mathrm{V}_\alpha}}{\tau_{\mathrm{life}}} \mathrm{d}z \ \ (< 0) \qquad \text{(A4)}$$

where τ_{life} implies the natural lifetime of an ionic vacancy.

The parallelepiped is finally enlarged to cover the whole polygon, and the mass balance is taken over the x-length of the polygon L. The resultant equation is expressed in a steady state as follows.

$$\bar{u} \int_0^L \frac{\partial}{\partial x} \int_0^h C_{\mathrm{V}_\alpha} \mathrm{d}z \, \mathrm{d}x = \frac{1}{\tau_{\mathrm{eff}}} \int_0^L \int_0^h C_{\mathrm{V}_\alpha} \mathrm{d}z\mathrm{d}x \qquad \text{(A5)}$$

where τ_{eff} is the effective extinction period of ionic vacancies, defined by

$$\frac{1}{\tau_{\mathrm{eff}}} \equiv \frac{1}{\tau_{\mathrm{col}}} + \frac{1}{\tau_{\mathrm{nano}}} + \frac{1}{\tau_{\mathrm{life}}} \qquad \text{(A6)}$$

Equation (A6) indicates that the effective extinction period is controlled by the smallest period, i.e., τ_{col}.

From Eq. (A5), we can represent the average concentration $\overline{C}_{\mathrm{V}_\alpha}$ over the polygon as follows.

$$\overline{C}_{\mathrm{V}_\alpha} = \frac{\tau_{\mathrm{eff}}}{L} \bar{u} \Delta C_{\mathrm{V}_\alpha} \qquad \text{(A7)}$$

where $\Delta C_{\mathrm{V}_\alpha}$ is denoted as the concentration difference between the inlet and the outlet of the polygon, i.e., the extinction concentration of the vacancy by collisions and lifetime in the reaction field.

$$\Delta C_{\mathrm{V}_\alpha} \equiv C_{\mathrm{V}_\alpha,\mathrm{elec}} - C_{\mathrm{V}_\alpha,\mathrm{wall}} \ \ (> 0) \qquad \text{(A8)}$$

where $C_{\mathrm{V}_\alpha,\mathrm{elec}}$ implies the concentration at the back edge of the electrodes (x= 0), and $C_{\mathrm{V}_\alpha,\mathrm{wall}}$ is the concentration at the inner wall (x = L).

In the same electrode system using the same current sweep, the parameters τ_{eff} and L are regarded constant, while the average velocity $\bar{u}$ induced by Lorentz force increases with the applied magnetic field. The more the average velocity $\bar{u}$ is enhanced, the shorter the staying period of vacancy in the reaction field becomes. This means that as $\bar{u}$ increases with the magnetic field, the chance of the extinction of the vacancy by collisions decreases, and the

concentration difference ΔC_{V_α} decreases. As a result, in a high magnetic field, Eq. (A7) leads to a constant vacancy concentration $\overline{C}_{V_\alpha}$.

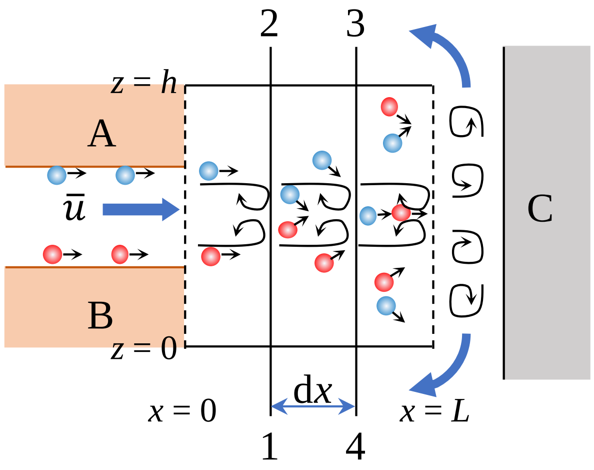

Fig. A1 A polygon of the reaction field defined in front of the inner wall of an electrolysis cell. A: cathode, B: anode, C: inner wall. $\bar{u}$: average velocity, h: z-height of the polygon, L: x-length of the polygon, blue circle: negative vacancy, red circle: positive vacancy.

Supplement B. Derivation of solvation energy stored by an ionic vacancy

In an electrolyte solution, ions are stabilized by solvation with the formation of ionic clouds liberating solvation energy. The internal energy U of the system composed of an ion and an ionic cloud at a constant temperature gives rise to the following equation in equilibrium.

$$\mathrm{d}U = \mathrm{d}w_{\mathrm{cloud}} + T\mathrm{d}S \ \ (= 0) \quad \text{(B1)}$$

where S denotes the entropy produced in the ionic cloud. w_{cloud} is the electric work of the ionic cloud emitting the solvation energy E_{sol}; so that this work is negative, $\mathrm{d}w_{\mathrm{cloud}} < 0$. As a result, from Eq. (B1), we obtain

$$T\mathrm{d}S = -\mathrm{d}w_{\mathrm{cloud}} \ \ (> 0) \quad \text{(B2)}$$

In the case of ions, it is concluded that the solvation energy released from the ionic cloud produces entropy in the solution.

An embryo vacancy emitted to a solution phase is also unstable and is immediately relaxed to a thermodynamic equilibrium state. The internal energy of a solvated vacancy is expressed by

$$\mathrm{d}U = \mathrm{d}w + T\mathrm{d}S \ \ (= 0) \tag{B3}$$

where w is the dynamic work of the ionic vacancy, where the electric work of the ionic cloud formation w_{cloud} is compensated by the expansion work of the vacancy core w_{core}

$$\mathrm{d}w = \mathrm{d}w_{\mathrm{core}} + \mathrm{d}w_{\mathrm{cloud}} \ \ (= 0) \tag{B4}$$

Substituting Eq. (B4) into Eq. (B3), we obtain

$$T\mathrm{d}S = 0 \tag{B5}$$

Namely, in solvation, an ionic vacancy does not produce entropy.

1. A reversible process of a solvated ionic vacancy

For a spherical vacancy core in an iso-dielectric solvent, due to rotational symmetry, the electric force by polarization acts normally on the tangential surface tension of the inner wall. In the following, we will therefore treat these two works w_{core} and w_{cloud} independently in a reversible process.

1.1 Expansion work w_{core} of a vacancy core

A vacancy core is expanded by the solvation energy donated from the ionic cloud, whose work is expressed by

$$\mathrm{d}w_{\mathrm{core}} = -\Delta P\mathrm{d}v \ \ (= \mathrm{d}E_{\mathrm{sol}}) \tag{B6}$$

where v is the volume of the vacancy core, and ΔP is the pressure difference between the external and internal pressures. E_{sol} is the solvation energy.

The volume is expressed by

$$v = \frac{4}{3}\pi r^3 \tag{B7}$$

where r is the radius of the vacancy core. The pressure is related to the surface tension of the inner wall, which is referred to as the Young-Laplace equation. In the special case of a spherical shape, it is relatively straightforward to represent that this equation may be written

$$P_{\mathrm{in}} - P_{\mathrm{ex}} = \frac{2\sigma}{r} \tag{B8}$$

where P_{in} is the internal pressure, which is zero in the present case of free space, and P_{ex} is the external pressure. The difference between P_{ex} and P_{in} is thus expressed by

$$\Delta P = -\frac{2\sigma}{r} \tag{B9}$$

where σ denotes the surface tension of a solvent. In the present case, it is equalized to that of water. As mentioned initially in this section, it is not changed by the polarization of the inner wall.

Subsequently, Eqs. (B7) and (B9) into Eq. (B6), and integrating the resulting equation from r = 0 to R_{V_α}, we obtain the dynamic work for the expansion of a vacancy core.

$$w_{\mathrm{core}} = 4\pi\sigma {R_{\mathrm{V}_\alpha}}^2 \tag{B10a}$$

where R_{V_α} denotes the radius of a solvated vacancy core. The solvation energy stored in the vacancy core is therefore expressed by

$$E_{\mathrm{sol}} = 4\pi\sigma {R_{\mathrm{V}_\alpha}}^2 \tag{B10b}$$

The theoretical molar excess heat of the pair annihilation of ionic vacancies with opposite charges in a redox reaction, i.e., the molar solvation energy stored in a pair of vacancies is written by

$$Q_{\mathrm{ann}} = 8\pi\sigma {R_{\mathrm{V}_\alpha}}^2 \tag{B11}$$

Supplement C. Determination of the radius of the vacancy core

1. Electric potential in an ionic cloud

An embryo vacancy released to a solvent phase first arranges polarized solvent molecules around it, attracting ions of opposite charge, and repelling ions of like sign. Then, the random thermal motion of ions and solvent molecules tends to counteract this electric effect and promotes a random distribution of ions, forming an ionic cloud around it. The balance of these competing effects of ionic species i in the ionic cloud can be expressed by a Boltzmann distribution of ionic molar concentration.

$$C_{\rm i} = C_{\rm i}(\infty)\exp\left(-\frac{z_{\rm i}F}{RT}\Phi\right) \quad \text{(C1)}$$

where $z_{\rm i}$ denotes the charge number of the ionic species i including sign, F is Faraday constant, R is the universal gas constant, T is the absolute temperature, and Φ is the electrostatic potential. $C_{\rm i}(\infty)$ is the bulk molar concentration of the species i. Far from the vacancy core, the potential Φ approaches zero, and consequently $C_{\rm i}$ approaches $C_{\rm i}(\infty)$. The potential Φ results not only from the polarized core but also from ions that are attracted toward or repelled by it.

In the present case, due to a spherical ionic cloud, the following simplified Poisson's equation in a radial coordinate r can be applied to the potential distribution.

$$\frac{1}{r^2}\frac{\rm d}{{\rm d}r}\left(r^2\frac{{\rm d}\Phi}{{\rm d}r}\right) = -\frac{\rho_{\rm E}}{\varepsilon} \quad \text{for } r > R_{\rm V_\alpha} \quad \text{(C2)}$$

where ε is the dielectric constant of the solvent, $R_{\rm V_\alpha}$ is the radius of the vacancy core, and $\rho_{\rm E}$ is the electric charge density at the distance r from the origin.

$$\rho_{\rm E} = F\sum_{\rm i} z_{\rm i}\,C_{\rm i} \quad \text{(C3)}$$

Assuming that the average molar energy of the electrostatic interaction is smaller than the average thermal molar energy RT, we derive the following approximation.

$$\exp\left(-\frac{z_{\rm i}F}{RT}\Phi\right) \approx 1 - \frac{z_{\rm i}F}{RT}\Phi \quad \text{(C4)}$$

Substituting Eq. (C4) into Eq. (C1), and inserting the resultant equation into Eq. (C3), we have

$$\rho_{\rm E} = -\frac{\varepsilon\Phi}{\lambda^2} \quad \text{(C5)}$$

where λ is the Debye length given by

$$\lambda = \left(\frac{\varepsilon RT}{F^2\sum_{\rm i} z_{\rm i}^2 C_{\rm i}(\infty)}\right)^{1/2} \quad \text{(C6)}$$

where the electroneutrality $\sum_{\rm i} z_{\rm i}\,C_{\rm i}(\infty) = 0$ of the bulk solution is used.

After these preparations, Eq. (C2) is solved under the following two boundary conditions. To the polarized inner wall of the core with the radius $R_{\rm V_\alpha}$, applying Gauss's formula, we obtain

$$4\pi r^2\left(-\frac{\mathrm{d}\Phi}{\mathrm{d}r}\right)=\frac{Q_{\mathrm{wall}}}{\varepsilon_0}\quad \text{for}\ \ r=R_{\mathrm{V_\alpha}} \tag{C7}$$

where Q_{wall} is the electric charge induced on the inner wall, ε_0 is the dielectric constant of free space. The inside of the core does not contain gas molecules since the diameter of the core is smaller than the mean free path of gas molecules. The potential Φ disappears far away from the center of the core.

$$\Phi=0 \quad \text{for}\ r\to\infty \tag{C8}$$

Under the boundary conditions Eqs. (C7) and (C8), Eq. (C2) is solved as follows.

$$\Phi(r)=\frac{Q_{\mathrm{wall}}}{4\pi\varepsilon_0 r}\frac{e^{\frac{(R_{\mathrm{V_\alpha}}-r)}{\lambda}}}{1+\frac{R_{\mathrm{V_\alpha}}}{\lambda}}\quad \text{for}\ \ r\geq R_{\mathrm{V_\alpha}} \tag{C9}$$

If there were no ionic cloud, the polarized inner wall would induce the following distribution.

$$\Phi_{\mathrm{wall}}(r)=\frac{Q_{\mathrm{wall}}}{4\pi\varepsilon_0 r}\quad \text{for}\ \ r\geq R_{\mathrm{V_\alpha}} \tag{C10}$$

Equation (C9) therefore reveals that the ions with opposite signs to the inner wall lower the magnitude of the potential and cause it to vanish rapidly at a large distance from the inner wall. This is the shielding effect of the ionic cloud, so the contribution of the ionic cloud to the potential is given by the difference between $\Phi(r)$ and $\Phi_{\mathrm{wall}}(r)$, i.e.,

$$\Phi_{\mathrm{cloud}}(r)=\frac{Q_{\mathrm{wall}}}{4\pi\varepsilon_0 r}\left\{\frac{e^{\frac{(R_{\mathrm{V_\alpha}}-r)}{\lambda}}}{1+\frac{R_{\mathrm{V_\alpha}}}{\lambda}}-1\right\} \tag{C11}$$

The contribution of the ionic cloud at r = $R_{\mathrm{V_\alpha}}$is expressed by

$$\Phi_{\mathrm{cloud}}\left(R_{\mathrm{V_\alpha}}\right)=-\frac{Q_{\mathrm{wall}}}{4\pi\varepsilon_0\lambda}\frac{1}{1+\frac{R_{\mathrm{V_\alpha}}}{\lambda}} \tag{C12a}$$

where Q_{wall} is the electric charge on the inner wall.

On the other hand, the potential at r = $R_{\mathrm{V_\alpha}}$ is derived from Eq. (C9). Since no electric charge exists inside the inner wall, in accordance with Gauss's formula, any electric field is also absent there. The potential of the inside of the core is thus kept constant.

$$\Phi_{\mathrm{core}}(r) = \frac{Q_{\mathrm{wall}}}{4\pi\varepsilon_0\lambda}\frac{1}{1+\frac{R_{\mathrm{V}_\alpha}}{\lambda}} \quad \text{for } r < R_{\mathrm{V}_\alpha} \tag{C12b}$$

2. Formation work w_{cloud} of an ionic cloud

To calculate the formation work of an ionic cloud, the electric charge of the inner wall Q_{wall} is reversibly increased, so that the solvation energy to produce E_{sol} is gradually released to the vacancy core.

$$dw_{\mathrm{cloud}} = \Phi_{\mathrm{cloud}}\left(R_{\mathrm{V}_\alpha}\right)\mathrm{d}Q_{\mathrm{wall}} \ (= -\mathrm{d}E_{\mathrm{sol}}) \tag{C13}$$

Substituting Eq. (C11) into Eq. (C13), and integrating the resultant equation from Q_{wall} = 0 to Q_{wall} = Q^*_{emb}, where Q^*_{emb} is the polarized electric charge transferring to an embryo vacancy, we obtain

$$w_{\mathrm{cloud}} = -\frac{{Q^*_{\mathrm{emb}}}^2}{8\pi\varepsilon_0\lambda}\frac{1}{1+\frac{R_{\mathrm{V}_\alpha}}{\lambda}} \tag{C14a}$$

The solvation energy donated from the ionic cloud to the vacancy core is thus given by

$$E_{\mathrm{sol}} = \frac{{Q^*_{\mathrm{emb}}}^2}{8\pi\varepsilon_0\lambda}\frac{1}{1+\frac{R_{\mathrm{V}_\alpha}}{\lambda}} \tag{C14b}$$

Due to electroneutrality, the absolute value of the total charge of the ionic cloud Q^*_{cloud}. is equal to that of Q^*_{emb}, but the opposite in sign.

$$Q^*_{\mathrm{cloud}} = -Q^*_{\mathrm{emb}} \tag{C15a}$$

Q^*_{emb} arises from the conservation of the electric charge in the electron transfer of an electrode reaction, so that we have

$$Q^*_{\mathrm{emb}} = ne^- \tag{C15b}$$

where *n* is the transferring charge number including the sign, and e^- is the unit charge. Substituting for E_{sol} from Eq. (B10b) in Eq. (C14b), we obtain the following equation for determining the radius of the vacancy core.

$$4\pi\sigma R_{V_\alpha}{}^2 = \frac{{Q^*{}_{\mathrm{emb}}}^2}{8\pi\varepsilon_0\lambda}\frac{1}{1+\frac{R_{V_\alpha}}{\lambda}} \qquad \text{(C16)}$$

Using Eqs. (B11) and (C16), we can calculate the theoretical molar excess heat of the pair annihilation of the vacancies with opposite signs in a redox reaction: First, we calculate the vacancy radius R_{V_α} from Eq. (C16). Then, substituting for the radius obtained from Eq. (C16) in Eq. (B11), we determine the theoretical molar excess heat Q_{ann}. In Fig. C1, the radius and theoretical molar excess heat of an ionic vacancy with two-unit charges created in a copper redox reaction are plotted against the molar concentrations of H_2SO_4 as a supporting electrolyte. As the concentration of the supporting electrolyte increases, the radius and the theoretical molar excess heat increase. This means that the solvation energy of ionic vacancy increases with the concentration of a supporting electrolyte.

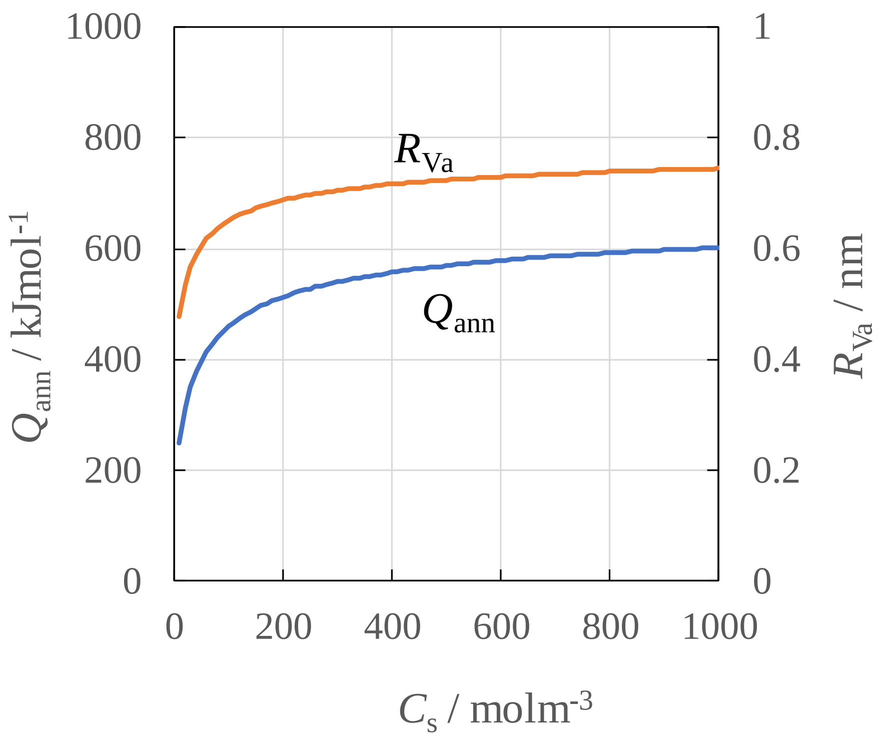

Fig. C1 Radius of a solvated ionic vacancy and theoretical molar excess heat of the pair annihilation of the vacancies with opposite two unit-charges in a copper redox reaction. R_{V_α}: theoretical vacancy radius, Q_{ann}: molar excess heat, C_{s}: the molar concentration of a supporting electrolyte H_2SO_4. Calculation data; unit electric charge: $e^- = 1.6021\times10^{-19}$ C, dielectric constant of free space:

ε_0 = 8.854×10^{-12} J^{-1}C^{2}m^{-1}, dielectric constant of water: ε = 6.954×10^{-10} J^{-1}C^{2}m^{-1}; Avogadro number: N_{AV} = 6.022×10^{23} mol^{-1}, Faraday constant: F = 96,500 C mol^{-1}, absolute temperature: T =298.0 K, Boltzmann constant: k_{B} = 1.38×10^{-23} JK^{-1}, surface tension of water at = 293.0 K: σ = 7.2×10^{-2} Jm^{-2}, transferring charge number: $n = 2$.

Supplement D. Raw data for Fig. 6

Some of the typical data for plotting Fig. 6 are exhibited with a simple explanation in the following.

1.1 The case of single c-type MHDEs

1) Ex. 1 of a single c-type MHDE

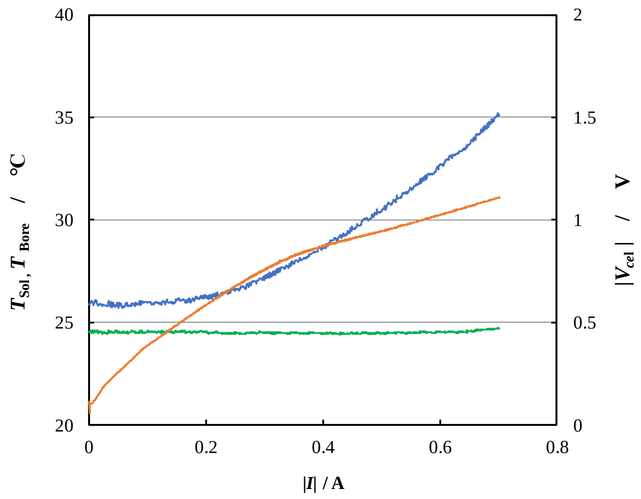

Fig. D1 Various temperature responses against the sweeping current. blue curve: solution temperature T_{sol}, green curve: bore temperature T_{Bore}, orange curve: cell voltage V_{cell}.

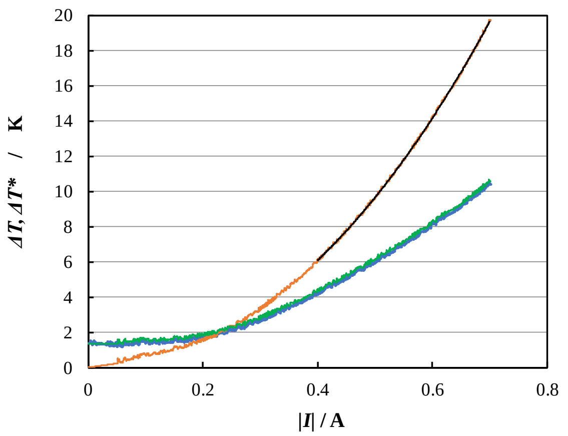

Fig. D2 Temperature differences between the solution and magneto-bore vs. sweeping current and application of the 3rd-order curve-fitting to it. blue curve: the temperature differences between the solution and magneto-bore ΔT, orange curve: the compensated temperature difference for dissipated heat ΔT^*, green curve: the compensated temperature difference for an initial state $\Delta T^*_{\mathrm{ini}}$, black curve: the 3rd-order curve-fitting $\Delta T^*_{\mathrm{fit}}$.

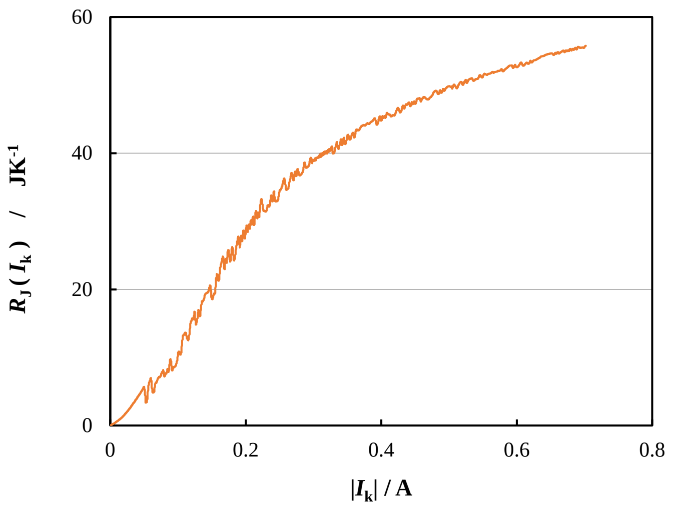

Fig. D3 $R_{\mathrm{J}}(I_{\mathrm{k}})$ vs. I_{k} plot.

$R_{\mathrm{J}}(I_{\mathrm{k}})$: Joule's heat capacity, I_{k}: k-th sampling data of I.

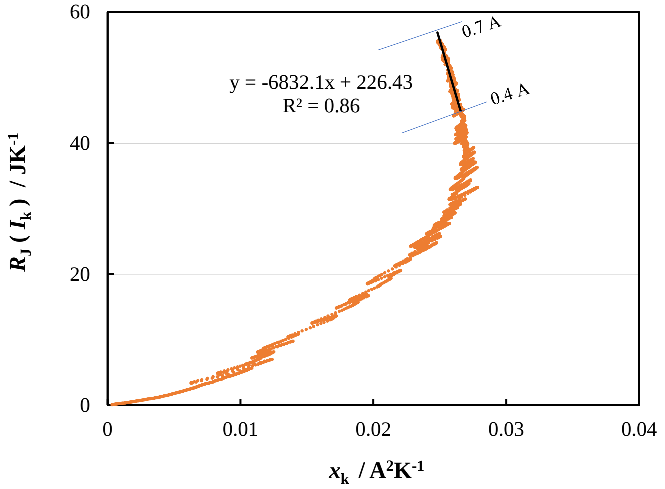

Fig. D4 $R_J(I_k)$-x_k locus.

$R_J(I_k)$: Joule's heat capacity, x_k: parameter defined by $I_k^{\ 2}/\Delta T^*$.

Table D1 Result of calculation

Sweep rate a / As-1	Sampling Δt / s	n	F / C·mol^{-1}	A_1	C_{sys} / J·K^{-1}	$\gamma_{ex}Q_{ex}$ / J·mol^{-1}	$\gamma_{ex}Q_{ex}$ / kJ·mol^{-1}	Remarks
2×10^{-4}	1	2	96,500	6,832	226	527,438	527	0.4-0.7A

2) Ex. 2 of a single c-type MHDE

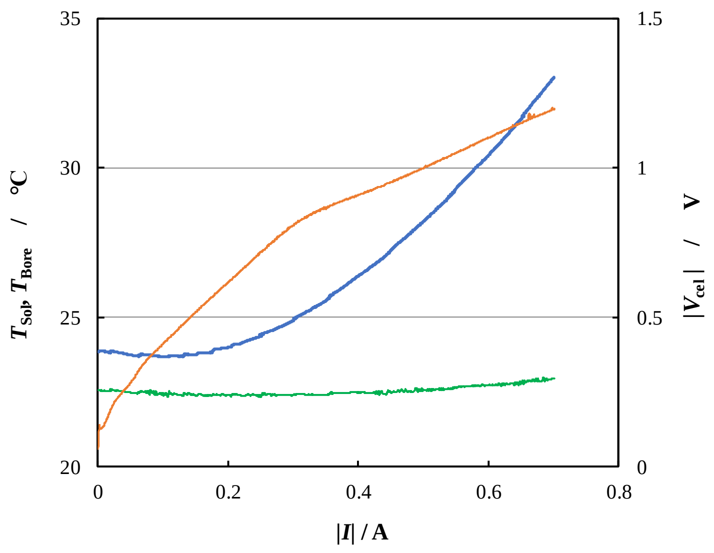

Fig. D5 Various temperature responses against the sweeping current. Blue curve: solution temperature T_{sol}, green curve: bore temperature T_{Bore}, orange curve: cell voltage V_{cell}.

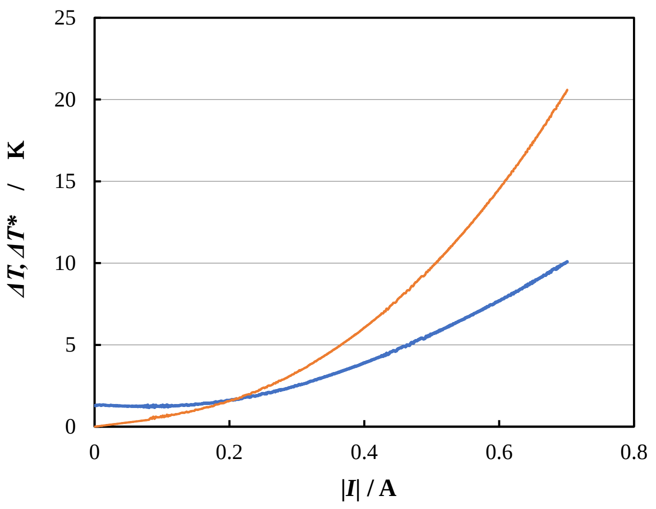

Fig. D6 Temperature differences between the solution and magneto-bore vs. sweeping current. Blue curve: temperature differences between the solution and magneto-bore ΔT, orange curve: the compensated temperature difference for dissipated heat ΔT^*.

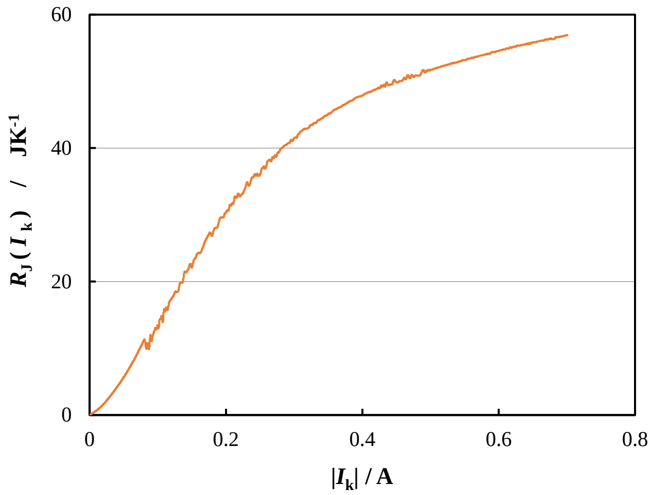

Fig. D7 $R_J(I_k)$ vs. I_k plot.

$R_J(I_k)$: Joule's heat capacity, I_k: k-th sampling data of I.

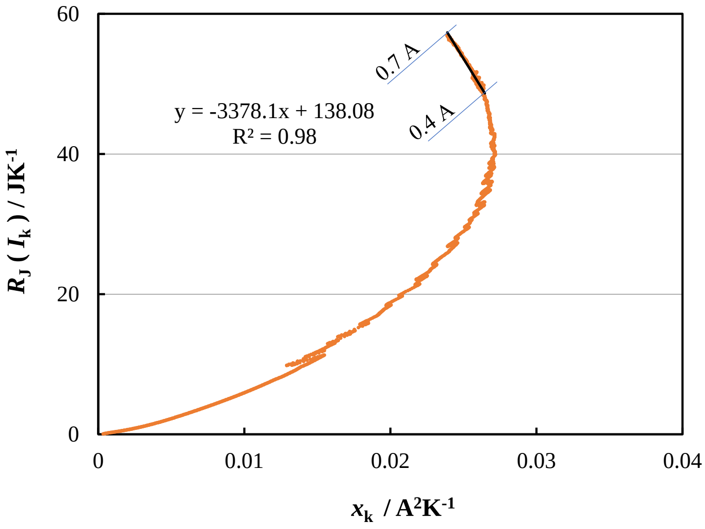

Fig. D8 $R_J(I_k)$ -x_k locus.

$R_J(I_k)$: Joule's heat capacity, x_k: parameter defined by $I_k^{\,2}/\Delta T^*$.

Table D2 Result of calculation

Sweep rate a / As-1	Sampling Δt / s	n	F / C·mol^{-1}	A_1	C_{sys} / J·K^{-1}	$\gamma_{ex}Q_{ex}$ / J·mol^{-1}	$\gamma_{ex}Q_{ex}$ / kJ·mol^{-1}	Remarks
2×10^{-4}	1	2	96,500	3,378	138	260,789	261	0.4-0.7A

1.2 The case of double c-type bipolar MHDEs

1) Ex. 1 of a double c-type bipolar MHDE

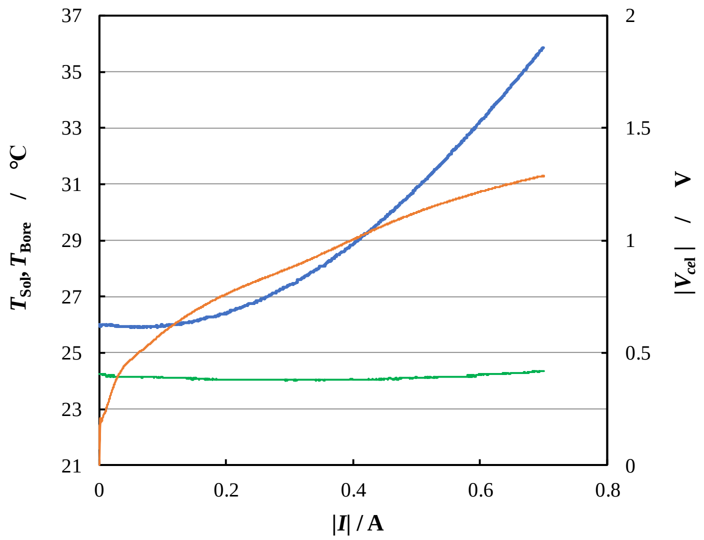

Fig. D9 Various temperature responses against the sweeping current. Blue curve: solution temperature T_{sol}, green curve: bore temperature T_{Bore}, orange curve, cell voltage V_{cell}.

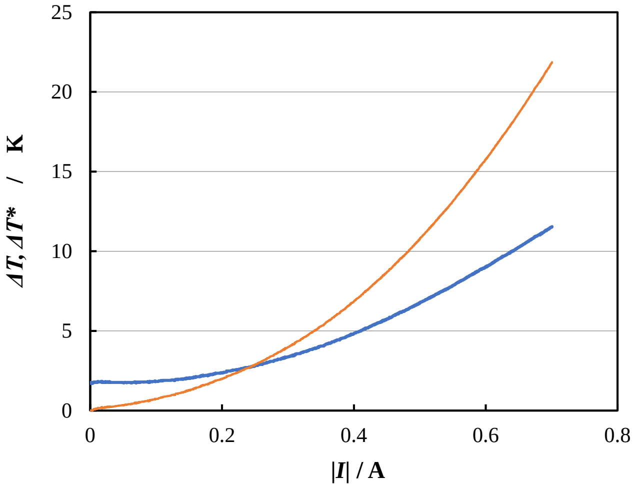

Fig. D10 Temperature differences between the solution and magneto-bore vs. sweeping current. Blue curve: temperature differences between the solution and magneto-bore ΔT, orange curve: the compensated temperature difference for dissipated heat ΔT^{*}.

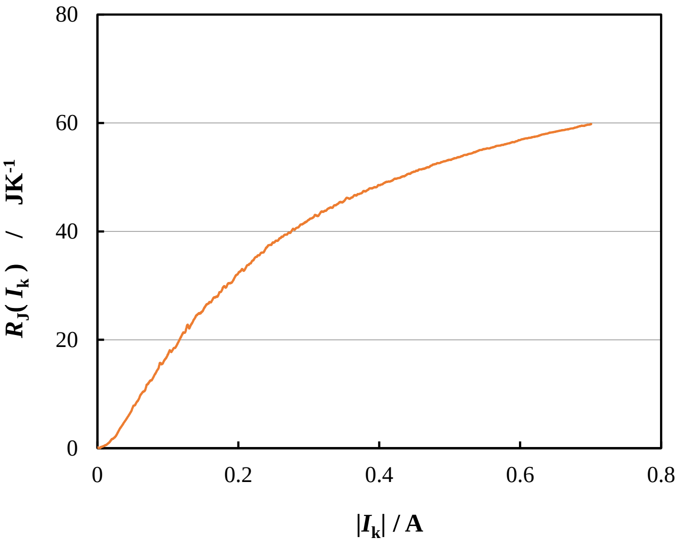

Fig. D11 $R_J(I_k)$ vs. I_k plot.

$R_J(I_k)$: Joule's heat capacity, I_k: k-th sampling data of I.

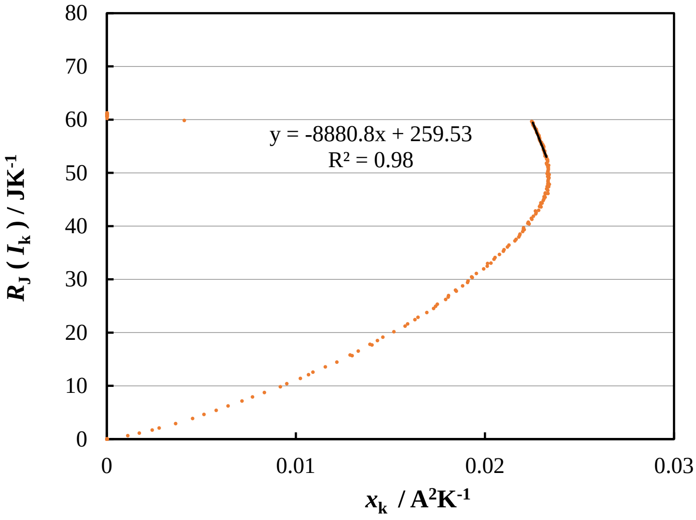

Fig. D12 $R_J(I_k)$-x_k locus.

$R_J(I_k)$: Joule's heat capacity, x_k: parameter defined by $I_k^2/\Delta T^*$.

Table D3 Result of calculation

Sweep rate a / As-1	Sampling Δt / s	n	F / $C \cdot mol^{-1}$	A_1	C_{sys} / $J \cdot K^{-1}$	$\gamma_{ex}Q_{ex}$ / $J \cdot mol^{-1}$	$\gamma_{ex}Q_{ex}$ / $kJ \cdot mol^{-1}$	Remarks
2×10^{-4}	1	2	96,500	8,881	260	685,598	686	

3) Ex. 2 of a double c-type bipolar MHDE

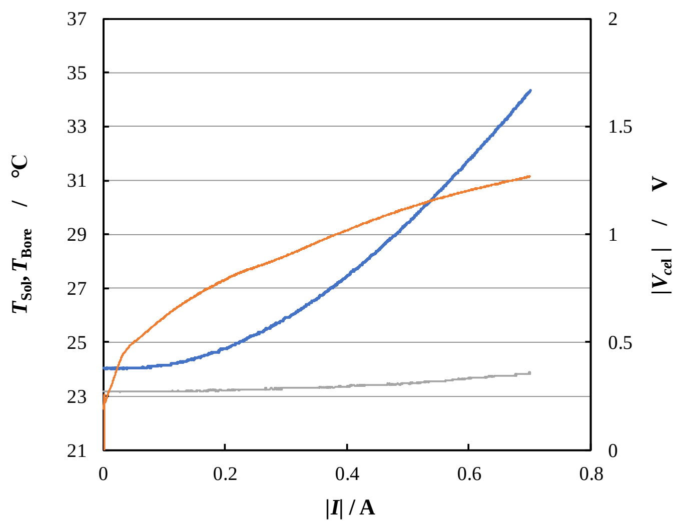

Fig. D13 Various temperature responses against the sweeping current.
Blue curve: solution temperature T_{sol}, green curve: bore temperature T_{Bore}, orange curve: cell voltage V_{cell}.

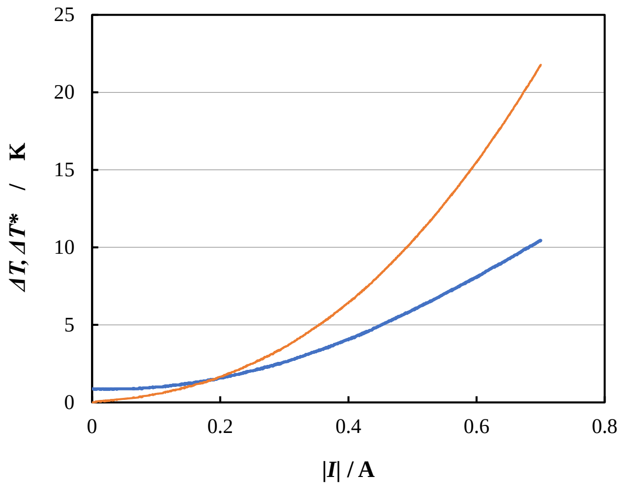

Fig. D14 Temperature differences between the solution and magneto-bore vs. sweeping current. Blue curve: temperature differences between the solution and magneto-bore ΔT, orange curve: the compensated temperature difference for dissipated heat ΔT^*.

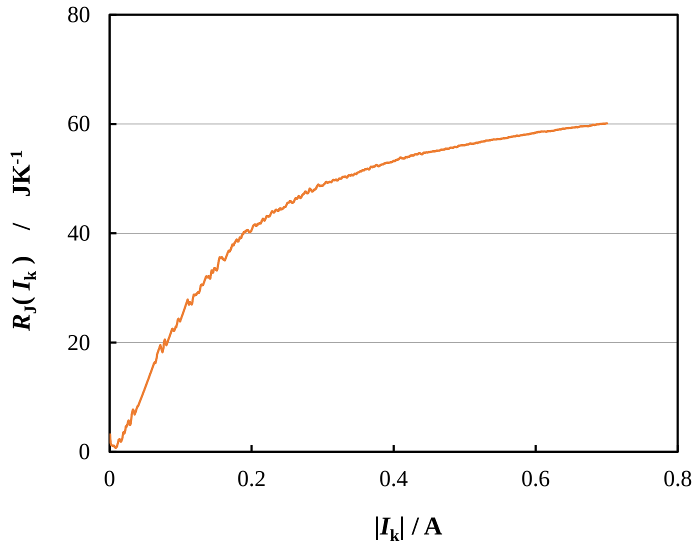

Fig. D15 $R_J(I_k)$ vs. I_k plot.

$R_J(I_k)$: Joule's heat capacity, I_k: k-th sampling data of I.

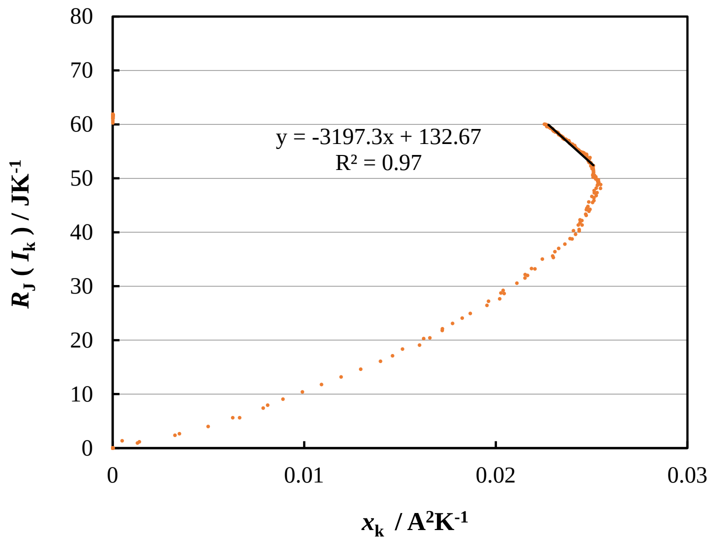

Fig. D16 $R_J(I_k)$-x_k locus.

$R_J(I_k)$: Joule's heat capacity, x_k: parameter defined by $I_k{}^2/\Delta T^*$.

Table D4 Result of calculation

Sweep rate a / As-1	Sampling Δt / s	n	F / C·mol^{-1}	A_1	C_{sys} / J·K^{-1}	$\gamma_{ex}Q_{ex}$ / J·mol^{-1}	$\gamma_{ex}Q_{ex}$ / kJ·mol^{-1}	Remarks
2×10^{-4}	1	2	96,500	3,197	133	246,832	247	

1.3 The case of double c-type bipolar MHDEs with diffuser

1) Ex. 1 of a double c-type bipolar MHDE with a diffuser

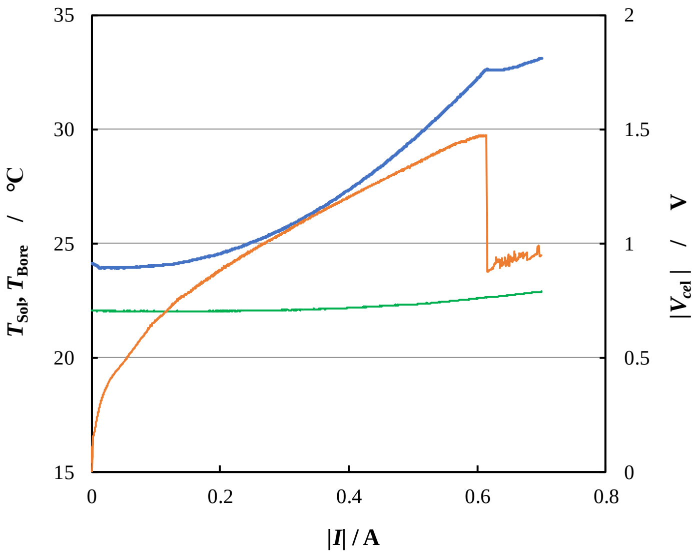

Fig. D17 Various temperature responses against the sweeping current.

Blue curve: solution temperature T_{sol}, green curve: bore temperature T_{Bore}, orange curve: cell voltage V_{cell}.

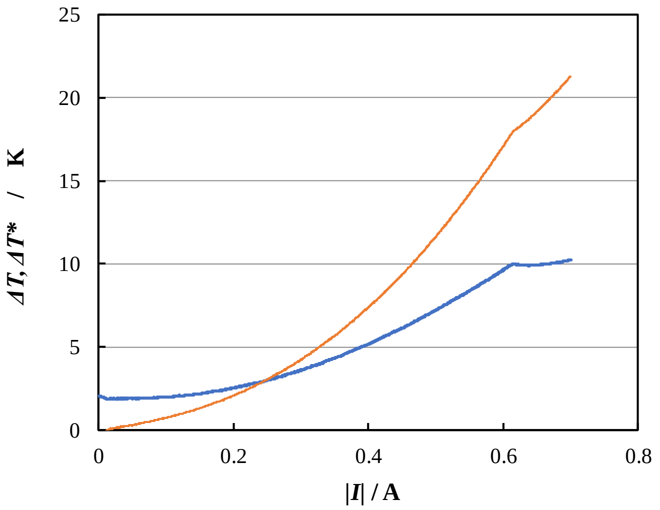

Fig. D18 Temperature differences between the solution and magneto-bore vs. sweeping current. Blue curve: temperature differences between the solution and magneto-bore ΔT, orange curve: the compensated temperature difference for dissipated heat ΔT^*.

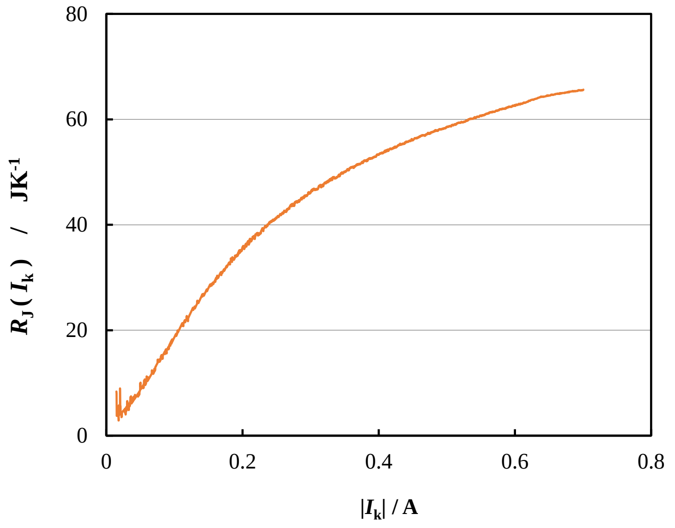

Fig. D19 $R_J(I_k)$ vs. I_k plot.

$R_J(I_k)$: Joule's heat capacity, I_k: k-th sampling data of I.

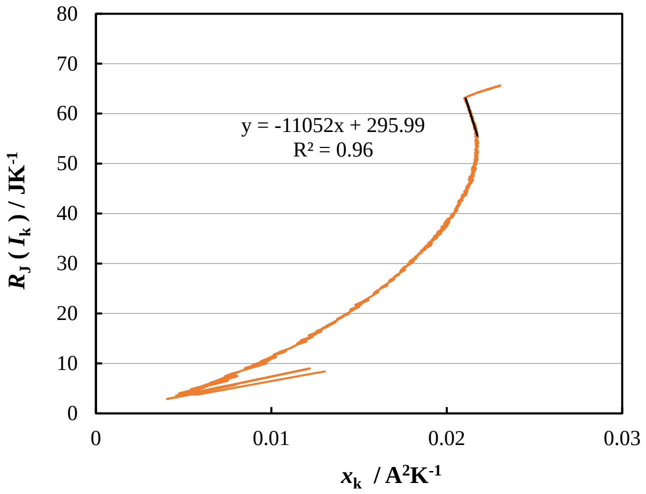

Fig. D20 $R_J(I_k)$-x_k locus.

$R_J(I_k)$: Joule's heat capacity, x_k: parameter defined by $I_k^2/\Delta T^*$.

Table D5 Result of calculation

Sweep rate a / As-1	Sampling Δt / s	n	F / C·mol^{-1}	A_1	C_{sys} / J·K^{-1}	$\gamma_{ex}Q_{ex}$ / J·mol^{-1}	$\gamma_{ex}Q_{ex}$ / kJ·mol^{-1}	Remarks
2×10^{-4}	1	2	96,500	11,052	296	853,214	853	

2) Ex. 2 of a double c-type bipolar MHDE with a diffuser

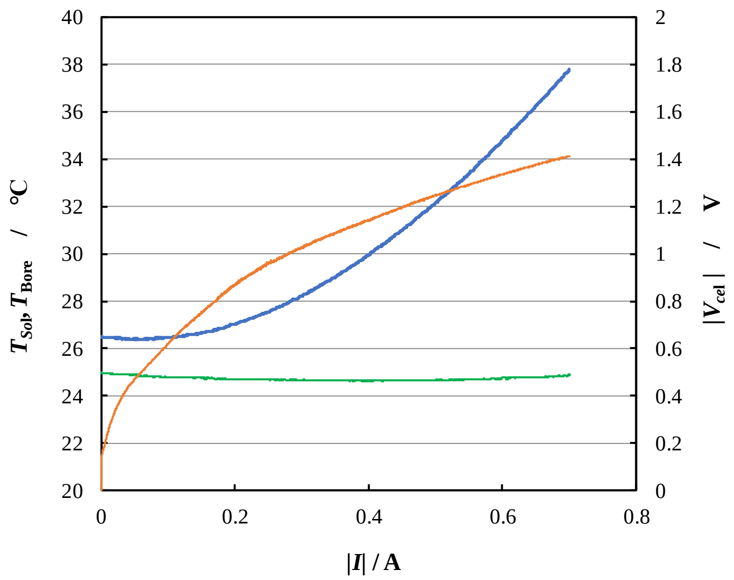

Fig. D21 Various temperature responses against the sweeping current. Blue curve: solution temperature T_{sol}, green curve: bore temperature T_{Bore}, orange curve: cell voltage V_{cell}.

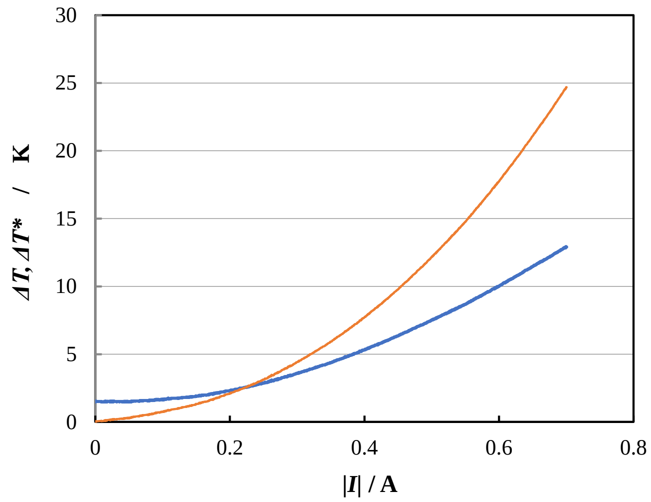

Fig. D22 Temperature differences between the solution and magneto-bore vs. sweeping current. Blue curve: temperature differences between the solution and magneto-bore ΔT, orange curve: the compensated temperature difference for dissipated heat ΔT^*.

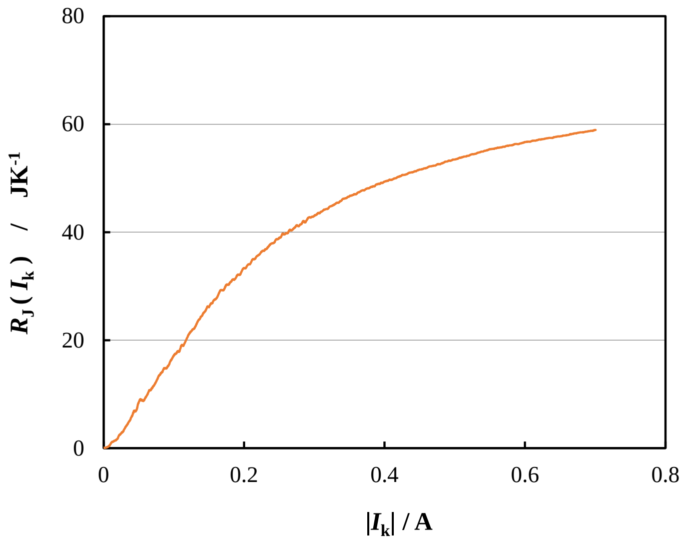

Fig. D23 $R_J(I_k)$ vs. I_k plot.

$R_J(I_k)$: Joule's heat capacity, I_k: k-th sampling data of I.

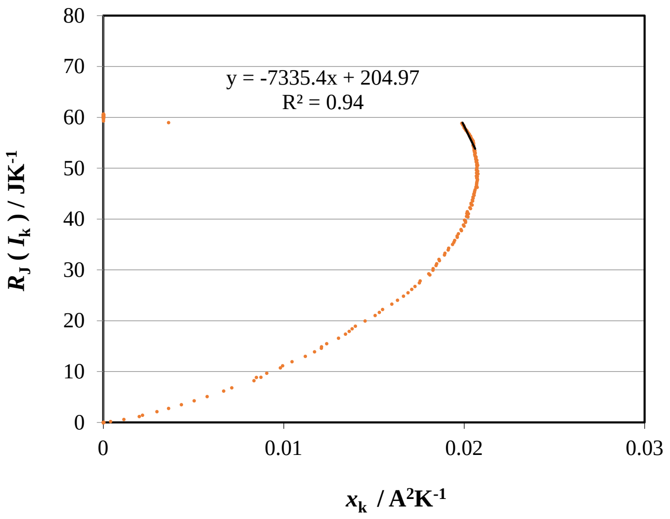

Fig.D24 $R_J(I_k)$ -x_k locus.

$R_J(I_k)$: Joule's heat capacity, x_k: parameter defined by $I_k{}^2/\Delta T^*$.

Table D6 Result of calculation

Sweep rate a / As-1	Sampling Δt / s	n	F / C·mol^{-1}	A_1	C_{sys} / J·K^{-1}	$\gamma_{ex}Q_{ex}$ / J·mol^{-1}	$\gamma_{ex}Q_{ex}$ / kJ·mol^{-1}	Remarks
2×10^{-4}	1	2	96,500	7,335	205	566,293	566	
